# Correction: Characterization and Functional Analysis of 4-Coumarate:CoA Ligase Genes in Mulberry

**DOI:** 10.1371/journal.pone.0157414

**Published:** 2016-06-08

**Authors:** Chuan-Hong Wang, Jian Yu, Yu-Xiang Cai, Pan-Pan Zhu, Chang-Ying Liu, Ai-Chun Zhao, Rui-Hua Lü, Meng-Jiao Li, Feng-Xiang Xu, Mao-De Yu

## Notice of Republication

This article was republished on June 1, 2016, to correct an error in the title. The word “Mulberry” was misspelled. Please download this article again to view the correct version. The originally published, uncorrected article and the republished, corrected article are provided here for reference.

## Supporting Information

S1 File Originally published, uncorrected article. (PDF)

S2 File Republished, corrected article. (PDF)

## References

[1] Wang C-H, Yu J, Cai Y-X, Zhu P-P, Liu C-Y, Zhao A-C, et al (2016) Characterization and Functional Analysis of 4-Coumarate:CoA Ligase Genes in Mulberry. PLoS ONE 11(5): e0155814 doi:10.1371/journal.pone.0155814 27213624 10.1371/journal.pone.0155814 PMC4877003

