# Supplementary material for: Characterization and Functional Analysis of 4-Coumarate:CoA Ligase Genes in Mulberry
Source: PLoS One. 2016 May 23;11(5):e0155814. doi: 10.1371/journal.pone.0155814 (PMC4877003; doi:10.1371/journal.pone.0155814)
Supplement: S1 File — (DOCX) [file pone.0155814.s001.docx]

**S1 File. The cDNA sequences of *Ma4CL* isolated from *M. atropurpurea* *cv*. Jialing No.40.**

>*Ma4CL1*

ATGGACGTCCCCCACCACCACCAAAAAGATCATGATCATCAAGAGGAATACATTTTCCGGTCCAAACTTCCCGACATCTACATCCCCAACCATCTTCCTCTCCACTCCTACTGCTTTGAGAACATCTCCAACTACCAAGACAAACCTTGTCTTATCAACGGATCCACAGGTGAGACCCACTCTTACGCCGATGTCGAGCTCACCGCGCGTAAAGTGGCGGCGGGGCTCGATGAGATGGGTATCAAACAAGGCGACGTGATTCTCCTCCTCCTCCAGAATTGCCCCGAATTCGTCTTCACCTTCCTCGCCGCCTCGTACATAGGAGCCATCAGCACCACCGCCAACTCTTTCTACACCCCGGCCGAGATCGCCAAGCAAGCAAAGGCGTCGAAGGCTAAACTTATCTTGACGGTTTCGACTTACGTCGACAAAGTCAAGAACTTCGCCGACGAAAATAGCGTCAAGATTGTGTGCATCGACGACGCCCCGCCCGAGGGGTGTCTCCACTTCTCGGAGCTTTCGAATGCCGACGAGTCCGCGATTCCGGCGGTGAAGATCAACCCAGACGACGTCGTTGCGCTGCCGTATTCATCGGGAACGACGGGGCTTCCTAAGGGGGTTATGCTGACGCACCGGGGACTCGTGACGAGTGTCGCTCAACAGGTTGATGGAGAGAATCCGAATTTGTACTTCCACAGCGAGGATGTGATCCTCTGCGTGCTTCCTCTTTTCCATATCTACTCCCTCAACTCGATCCTGCTCTG

>*Ma4CL2*

ATGGCCGATTCAGCGGATCAGAAAGACATTATATTTCGCTCAAAACTCCCCGATATCTACATCCCCAAACATCTCCCTCTCCACACTTACTGCCTCTCCAACAAATACCAGCAGCGCTCTCGTCCCTGCCCCATCAACGGCCCCACCGGAGATGTCTACACCTACGCCGACGTCGACCTCAAAGCCCGAAAGGTCGCCGCGGGCCTCCACAGGCTCGGCGTGCGAAAGGGCGATGTTGTCATGGTTGCTCTTCCCAATTCGCCGGAATTCGTCCTGACCTTCTTGGGAGCGTCTTATCGCGGCGCCATGACGACGGCGGCCAACCCCTTTGTCACCTCCGCCGAGATTCTAAAACAAGCTAGGGCTTCCGACGCGAAGCTCGTCGTCACGCAAGCTTGTTACTACGACAAGGTTAAGGATTTGAAACTTGACAATGAAGACGACAACATCACGATCGTGTGCGTGGACACCCCGGTCCCCGAAGGTTGTTTGCATTTCTCCGAGCTGACTAATTCCGACGAGAACGCCTTGCCGGCGGTGGATATCAGCCCCGACGACGTCGTCGCGCTGCCGTACTCTTCGGGCACGACGGGGCTGCCGAAGGGGGTGATGCTGACGCACAAGGGGCTGGTGACGAGCGTGGCGCAGCAGGTGGATGGGGAGAATCCCAACTTGTATTACGGCGAGGACGACGTCGTCTTGTGCGTGCTGCCACTGTTCCACATATACTCGCTGAACTCGGTGCTGCTGTGCGGGCTGAGAGCTGGAGCAACGATCGTGATCATGCCCAAGTTCGAGATCGGGTCGCTGCTGGAGCTGATTCAGAAGTACAAGATCAGCGTTGTGCCGATTGTGCCGCCGATCGTGATGGCGATAGCCAAGTACCCGAGTCTGGATAAATATGACTTGTCGTCGCTGAGGGTTCTGAAGAGCGGAGGAGCACCGCTGGGGAAGGAGCTTGAAGACACGGTGAGAGCCAAGTTTCCCAACGTCACGCTTGGACAGGGGTATGGAATGACGGAGGCAGGGCCAGTACTAACGATGTCGTTGGCGTTTGCTAAGGAGCCCATGGAAGTAAAACCAGGGGCATGTGGAACTGTCGTAAGGAATGCAGAGCTCAAAATTGTTGATCCTGAAACCGGCTCCTCCCTCCCACGCAACCAGCCTGGCGAGATTTGCATTAGAGGTGATCAGATCATGAAAGGTTATCTCAATAATCCGGAGGCTACAAAGAACACAATAGACAAAGAAGGGTGGCTACACACAGGTGATATTGGGTTGGTGGATGATGATGAGGAGCTCTTCATTGTGGATCGCTTAAAAGAGCTCATCAAATACAAAGGCTTTCAGGTGGCCCCCGCAGAGCTTGAAGCCTTGCTTCTCACTCACCCTCACATCTCTGATGCCGCTGTTGTCCCTATGAAAGATGGAGCAGCTGGAGAGGTTCCTGTTGCATTTGTGGTGAGGGCAAATGGATCTCAGATCACTGAGGACGAAATCAAACAATTTGTTTCTAAACAGGTTGTGTTCTACAAAAGAATAAATCGAGTAATTTTCATCGAGGCCATTCCAAAGTCTCCATCAGGGAAAATCTTGCGCAAGGACTTGAGAGCGAAACTTGCCGCTGGCTTTCCTAACTAA

>*Ma4CL3*

ATGATTTCCGTAGCTAACAACTCCTCCATTGAAACCCACTCTCCCAACGACAATGCTAGTACTACAACTGCTAGTCATGTTTTCAAATCCAAATTACCAGACATCACCATCTCCAACAACATCCCTCTCCACGCATACTGCTTCGAGCGCTTGTCGGAGTTCGCGGACAGGCCATGTATCATCTCTGGCTCCACCGGCAAGACCTACACTTACGCCGAAACCCACCACATCACCCGAAAGATCGCGGCAGGACTCGCTCGTCTCGGTGTCAAGAAAGGAGACGTCTGCATGATCCTCCTCCAGAATTGCCCCGAGTTCGTCTTCTCCTTCATGGCCACTTCCATGCTCGGCGCCGTCGCTACCACCGCCAATCCTTTCTACACCGCAGCCGAGATCTGCAAACAATTCACCGCCGCCAACGTCAAGCTCATCATCACCCAATCCCAATACGTCGACAAGCTCCGCGACGACCCCGCCGCCGCGGACGACAAGAAGTTCCCGAAAATGGGAGAAGACTTCAAGGTCATCACCGTCGACGACCCGCCGGAGGATTGCCTACACTTCTGGTCACTACTCCTGAAAAGGAATAAAAAGACAACGGAAACGATCCAATATCAGTCTGAAGAAGAAGAAGAAGAAGAAGAAGAAGAAGATGATGATGATCAATTATTAGATGCGATAAAAATCAGCGCGGACGATCCAGTGGCACTCCCATTCTCCTCCGGAACCACGGGGCTTCCCAAGGGAGTCATCCTAACTCACAAGAGTCTGATAACAAGCGTGGCTCAGCAAGTCGACGGAGAAAACCCTAACCTCCATCTGACGACACAGGACGTGTTTCTATGCGTTCTGCCGCTGTTCCACATATTCTCGATGAACAGCGTGATGCTAAACTCGCTCCGCTCCGGCGCGGCTGTGCTGCTGATGCCTAAGTTCGAGATCGGGACGTTGCTGGAGCTGATACAGAAGCACCGGGTGTCGATAGCGGCGGTGGTGCCCCCGCTGGTGCTGGCGCTGGCGAAGAACCCGAAGGTGGCGGAATACGATCTCAGCTCCATTCGGATTGTGCTTTCCGGGGCGGCTCCGCTGGGGAAGGAGCTTCAGGACGCGCTCAGGAGCAGAGTTCCTCGGGCAATTCTTGGCCAGGGTTATGGAATGACTGAGGCAGGACCAGTGATATCAATGTCGTTGTCGTTTGCAAAGCAGCCATTTCCACCCAAGTTAGGCTCATGCGGTTGTGTGGTTAGGAATGCAGAGCTCAAGGTTATTGACCCTGAAACTGGCTCTTCTCTTGGCTACAACCAGCCTGGAGAGATTTGCATTCGAGGATCCCAAATTATGAAAGGGTATTTGAACGATGACCAGGCCACGGCGGGCACCATAGATGTTGAGGGCTGGCTTCATACCGGAGACATAGGTTACGTTGACGATGATGAAGAAATTTTCATTGTTGATAGAGTTAAGGAGCTTATCAAATTCAAAGGTTTTCAGGTACCACCAGCTGAGCTTGAGTCTCTTCTTGTAGGCCATCCTTCAATAGCAGACGCAGCTGTAGTCCCGCAAAAAGACGACGCCGCCGGCGAAGTTCCCGTAGCGTTTGTTGTTCGATCTAACAGCGGTCTTACAGAAGAGGCTGTAAAAGAATTCATAGCAAAACAGGTGGTGTTTTACAAGAAATTACACAAGGTGTACTTCGTCCATGCAATTCCAAAGTCCCCATCTGGAAAGATCCTAAGAAAGGACCTAAGAGCCAGGCTTGCTACAGCTTCCCCTTTGAGCTGA

>*Ma4CL4*

ATGGATCCTGCGCATCATGATCACAAAGAAGAAAATGGCCACGAACAGTTCATTTTTCGCTCAAAACTCCCTAACATTCACATCCCCAACCACCTTCCTCTCCACACCTACTGCTTTCAAAACATCTCCAAATTCAAAGATCGCCCATGTCTGATCAACGCCTCCACCGGCGACACCCACACCTACGCCGACGTCGACCTCGCGGCGCGCCGAGTCGCCGCGGGGCTCCACGGCTTGGGTATCCGGAAGGGTGACGTCGTCATGCTCTTGCTCCATAACTGTCCCGAATTCGTCTTCGCCTTCCTCGGCGCTTCGCATCTCGGAGCCGTCACCACCGCCGCCAACCCGCTCTTCACCCCCGCCGAGGTCGCGAAGCAGGCGATGAGCTCCAAAGCCAAACTCATAATCACTTTCTCGGCTTACGTAGAGAAGGTGGAGGAACTAGCCCTGGAGAAAAATCACAACGGTTTGAGGATCGCGTGCATCGACGCGCCGCCCGAGGGGTGCGTGGATTTCTCGGAGTTGATGCTTGCCGACGAGAAGGAAAAACCGACGGTGGAGATCGAACCGGACGATGTCGTTGCACTGCCGTATTCTTCGGGCACGACGGGGCTACCGAAAGGGGTGATGGTGACACACAAGGCGCTTGTGACGAGCGTGGCGCAGCAAGTCGATGGTGAAAACCCTAACTTGTACTACCGCATCGAGGACGTGATTCTCTGCGTGCTTCCTTTGTTCCACATTTACGCTCTAAGCTCGATTTTGCTATGCGGATTACGGGTCGGATCGGCGATCTTGATCATGCCGAGGTTTGAGATCGGCAAGTTGTTAAAATTAGTGGAGTGGTATAAGGTGACGGTAACACCATTTGTTCCGCCGATACTCTTGTCGATCGCGAAAAACCCCGATCTCGACCGATACGATTTGTCGTCGATTCGGATGATTATTACGGGCGGAGCGCCAATGGGGAAGGAGCTTGAGGAGGCCGTCAAGGATAAGCTTCCTCATGCCAAGCTTGGGCAGGGTTATGGGATGACAGAAGCGGGATCGGTGTCGATGTGCTTGGCATTTGCCAAGGAACCCTTCCCCATAAAGTCTGGCACTTGCGGCACCATCGTGAGGAATGCCGAGATGAAGATAATCGACCCCAACACCGGCTTGTCACTTCCACAAAACCGAGCTGGTGAGATTTGCATCAGAGGTAGCCAAATCATGAAAGGTATCATATACACATCACTTTAA
