# Supplementary material for: Characterization and Functional Analysis of 4-Coumarate:CoA Ligase Genes in Mulberry
Source: PLoS One. 2016 May 23;11(5):e0155814. doi: 10.1371/journal.pone.0155814 (PMC4877003; doi:10.1371/journal.pone.0155814)
Supplement: S2 File — The purified Ma4CL3 protein was analyzed by MALDI-TOF/TOF MS, and the results showed that the purified protein peptides completely matched the predicted peptides (PDF) [file pone.0155814.s002.pdf]

Rank	Protein Name	Accession No.	Protein MW	Protein PI	Pep. Count	Protein Score	Protein Score C. I. %	Intensity Matched	Total Ion Score	Total Ion C. I. %	Confirmed
1	4-coumarate--CoA ligase 3 [Morus notabilis]	gi|587838458	65230.9	6.05	14	617	100	32.806	561	100	

Peptide Information

Calc. Mass	Obsrv. Mass	± da	± ppm	Start Seq.	End Seq.	Sequence	Ion Score	C. I. %	Modification	Rank	Result Type
844.4523	844.4637	0.0114	13	368	374	ELQDALR					Mascot
1007.4761	1007.4838	0.0077	8	410	418	LGSCGCVVR			Carbamidomethyl (C)[4,6]		Mascot
1152.5896	1152.6001	0.0105	9	347	356	VAEYDLSSIR					Mascot
1152.5896	1152.6001	0.0105	9	347	356	VAEYDLSSIR	63	99.998			Mascot
1444.7432	1444.7574	0.0142	10	525	538	DDAAGEVPVAFVVR					Mascot
1444.7432	1444.7574	0.0142	10	525	538	DDAAGEVPVAFVVR	131	100			Mascot
1561.0088	1560.9777	-0.0311	-20	328	343	VSIAAVVPPLVLALAK					Mascot
1652.9482	1652.871	-0.0772	-47	314	327	FEIGALLELVQKHR					Mascot
1837.9114	1837.9277	0.0163	9	59	75	LSEFADRPCIISGSTGK			Carbamidomethyl (C)[9]		Mascot
1837.9114	1837.9277	0.0163	9	59	75	LSEFADRPCIISGSTGK	75	100	Carbamidomethyl (C)[9]		Mascot
1973.0227	1973.0139	-0.0088	-4	231	250	ISADDPVALPFSSGTTGL PK					Mascot
2273.1384	2273.1592	0.0208	9	40	58	LPDITISNNIPLHAYCFER			Carbamidomethyl (C)[16]		Mascot
2305.113	2305.1296	0.0166	7	424	444	VIDPETGSSLGYNQPGEI CIR			Carbamidomethyl (C)[19]		Mascot
2305.113	2305.1296	0.0166	7	424	444	VIDPETGSSLGYNQPGEI CIR	171	100	Carbamidomethyl (C)[19]		Mascot
2397.2161	2397.2207	0.0046	2	183	202	VITVDDPPEDCLHFWSLL LK			Carbamidomethyl (C)[11]		Mascot
2428.2251	2428.2163	-0.0088	-4	380	403	AILGQGYGMTEAGPVISM SLSFAK					Mascot
2488.2654	2488.2815	0.0161	6	38	58	SKLPDITISNNIPLHAYCF ER			Carbamidomethyl (C)[18]		Mascot
2488.2654	2488.2815	0.0161	6	38	58	SKLPDITISNNIPLHAYCF ER	121	100	Carbamidomethyl (C)[18]		Mascot
2807.2588	2807.343	0.0842	30	207	230	KTETIQDQSEEEEDDDD QLLDAIK					Mascot
