# Supplementary material for: Characterization and Functional Analysis of 4-Coumarate:CoA Ligase Genes in Mulberry
Source: PLoS One. 2016 May 23;11(5):e0155814. doi: 10.1371/journal.pone.0155814 (PMC4877003; doi:10.1371/journal.pone.0155814)
Supplement: S3 File — All of the Mn4CL genes were predicted to encode a family of proteins that includes Mn4CL1 with 646 amino acids, Mn4CL2 with 546 amino acids, Mn4CL3 with 595 amino acids, and Mn4CL4 with 407 amino acids. (DOCX) [file pone.0155814.s003.docx]

**S3 File. The predicted Mn4CLs protein sequences.**

>Mn4CL1

MDVPHHHQKDHDHQEEYIFRSKLPDIYIPNHLPLHSYCFENISNYQDKPCLINGSTGETHSYADVELTARKVAAGLDEMGIKQGDVILLLLQNCPEFVFTFLAASYIGAISTTANSFYTPAEIAKQAKASKAKLILTVSTYVDKVKNFADENSVKIVCIDDAPPEGCLHFSELSNADESAIPAVKINPDDVVALPYSSGTTGLPKGVMLTHRGLVTSVAQQVDGENPNLYFHSEDVILCVLPLFHIYSLNSILLCGLRVGAAILIMQKFEINKLLELVEKYKVTIAPFVPPIVLAIAKSPDLHRYDLSSIRMVMSGGAPMGKDLEEAVKDKLPHAKLGQGYGMTEAGPVLSMCLAFAKEPFPIKSGACGTVVRNAEMKIIDPDTGASLPRNQAGEICIRGSQIMKGYVNDPEATKATIDERGWLHTGDIGYIDDDDELFIVDRLKELIKYKGFQVAPAELEAMLIAHPNISDAAVVPMNDEAAGEIPVAYVVRSNGSKITENDIKKYISDQVVFYKRIGKVIFIDKIPKSPSGKILRKDLRARLTAEHENNTQHTTVHLFTTGNLKASIVIAPNAAAPTVTGPPSRSHHRRSSPPQALAITIPHRRGPHYRGLTVATPQRRSLPSTISACNAGKPASSTISISNAV*

>Mn4CL2

MADSADQKDIIFRSKLPDIYIPKHLPLHTYCLSNKSQQRSRPCLINGPTGDVYTYADVDLKARKVAAGLHKLGVRKGDVVMVVLPNSPEFVLTFLGASYRGAMTTAGNPFFTSAEILKQARASDAKLVVTQACYYDKVKDLKLDNEDDNITIVCVDAPVPEGCLHFSELTNSDENALPAVDISPDDVVALPYSSGTTGLPKGVMLTHKGLVTSVSQQVDGENPNLYYGEDDVVLCVLPLFHIYSLNSVLLCGLRAGATVVIMPKFEIGSLLELIQKYKISVVPIVPPIVLAIAKYPSLDKYDLSSLRVLKSGGAPLGKELEYTVRAKFPNVTLGQGYGMTEAGPVLTMSLAFAKEPMEVKPGACGTVVRNAELKIVDPETGSSLPRNQPGEICIRGDQIMKGYLNNPEATKNTIDKEGWLHTGDIGLVDDDEELFIVDRLKELIKYKGFQVAPAELEALLLTHPHISDAAVVPMKDEAAGEVPVAFVVRANGSQITEDEIKQFVSKQVVFYKRINRVIFIEAIPKSPSGKILRKDLRAKLAAGFPN*

>Mn4CL3

MISVANNSSIETQQQTPAERISPNDNSSTTTASHVFKSKLPDITISNNIPLHAYCFERLSEFADRPCIISGSTGKTYTYAETHLITRKIAAGLVRLGVKKGDVCMILLQNCPEFVFSFMAASMIGAVATTANPFYTAAEICKQFTLANAKLIITQSQYVDKLRDAPAAADDKKLPKMGEDFKVITVDDPPEDCLHFWSLLLKRNKKKTETIQDQSEEEEDDDDQLLDAIKISADDPVALPFSSGTTGLPKGVILTHKSLITSVAQQVDGENPNLHLTTQDVFLCVLPLFHIFSMNSVMLNSLRSGAAVLLMHKFEIGALLELVQKHRVSIAAVVPPLVLALAKNPKVAEYDLSSIRIVLSGAAPLGKELQDALRSRVPRAILGQGYGMTEAGPVISMSLSFAKQPFPPKLGSCGCVVRNAELKVIDPETGSSLGYNQPGEICIRGSQIMKGYLNDDKATAGTIDVEGWLHTGDIGYVDDDQEIFIVDRVKELIKFKGFQVPPAELESLLVSHPSIADAAVVPQKDDAAGEVPVAFVVRSNSGLTEEAVKEFIAKQVVFYKKLHKVYFVHAIPKSPSGKILRKDLRARLATASPLS*

>Mn4CL4

MDPAHHDHKEENGHEQFIFRSKLPNIHIPNHLPLHTYCFQNISKFKDRPCLINASTGDAHTYADVDLAARRVAAGLHGLGIRKGDVVMLLLHNCPEFVFAFLGASHLGAVTTAANPLFTPAEVAKQATNSKAKLIITFSAYVGKVEELALEKNHNGLRIACIDAPPEGRVDFSEVMLADEKEKPTVEIEPDDVVALPYSSGTTGLPKGVMVTHKALVTSVAQQVDGENPNLYYRSEDVILCVLPLFHIYALNSILLCGLRVGSAILIMPRFEIGKLLKLVEWYKVTVTPFVPPILLSIAKNPDLDRYDLSSIRMIITGGAPMGKELEEAVKDKLPHAKLGQGYGMTEAGSVSMCLAFAKEPFPIKSGACGTVVRNAKIIDPNTVLSLPRNRAGEICIRGSQIMKGIIYTSL*
