# Supplementary material for: Characterization and Functional Analysis of 4-Coumarate:CoA Ligase Genes in Mulberry
Source: PLoS One. 2016 May 23;11(5):e0155814. doi: 10.1371/journal.pone.0155814 (PMC4877003; doi:10.1371/journal.pone.0155814)
Supplement: S1 Table — (DOCX) [file pone.0155814.s004.docx]

**S1 Table The primers were used to isolate the *Ma4CL* genes in *M. atropurpurea cv. Jialing* No.40.**

| Gene | Primer (5' to 3') |
| --- | --- |
| *Ma4CL1* | Forward: ATGGACGTCCCCCACCACCACCAA |
|  | Reverse: CAGAGCAGGATCGAGTTGAG |
| *Ma4CL2* | Forward: ATGGCCGATTCAGCGGATCAGAA |
|  | Reverse: GTTAGGAAAGCCAGCGGC |
| *Ma4CL3* | Forward: ATGATTTCCGTAGCTAACAA |
|  | Reverse: GCTCAAAGGGGAAGCTGTA |
| *Ma4CL4* | Forward: ATGGATCCTGCGCATCATGATCA |
|  | Reverse: AAGTGATGTGTATATGATACCTTTC |
