# Supplementary material for: Characterization and Functional Analysis of 4-Coumarate:CoA Ligase Genes in Mulberry
Source: PLoS One. 2016 May 23;11(5):e0155814. doi: 10.1371/journal.pone.0155814 (PMC4877003; doi:10.1371/journal.pone.0155814)
Supplement: S3 Table — (DOCX) [file pone.0155814.s006.docx]

**S3 Table Oligonucleotides for vector construction in this study.**

| Name | Oligonucleotide sequence (5'–3')*^a^* | Target vector | | Relevant  characteristic |
| --- | --- | --- | --- | --- |
| pET4CL3-F | CCCAAGCTTCCATGATTTCCGTAGCTAACAA | | pET*4CL3* | *Hin*d III |
| pET4CL3-R | CCGCTCGAGGCTCAAAGGGGAAGCTGTAG | |  | *Xho* I |
| pETDuet4CL3-F | GGGTACCATGGAATCGGTCAAAGAATTCC | | pETDuet-*4CL3*-*STS* | *Kpn* I |
| pETDuet4CL3-R | CCTCGAGTTATGCAGAGTGATCAATTGCA | |  | *Xho* I |
| pETDuetSTS-F | GCTGCAGATGGAATCGGTCAAAGAATTCCG | |  | *Xho* I |
| pETDuetSTS-R | CAAGCTTCTATGCAGAGTGATCAATTGCAACG | |  | *Hin*d III |
| pETSTS-F1 | CCCGGATCCATGGAATCGGTCAAAGAATTCCG | | pET*STS*-*4CL3* | *Bam*H I |
| pETSTS-R1 | ***ACCCGATCCGGAAGA***CTATGCAGATGATCAATTG | |  | linker |
| pET4CL3-F1 | ***TCATCTTCCGGATCGGGT***ATGATTTCCGTAGCTAACAA | |  |  |
| pET4CL3-R1 | CCTCGAGGCTCAAAGGGGAAGCTGTAG | |  | *Xho* I |
| pET4CL3-F2 | CCCGGTACCCCATGATTTCCGTAGCTAACAA | | pET*4CL3*-*STS* | *Bam*H I |
| pET4CL3-R2 | ***ACCCGATCCGGAAGATGA***GCTCAAAGGGGAAGCTGTAG | |  | linker |
| pETSTS-F2 | ***TCATCTTCCGGATCGGGT***ATGGAATCGGTCAAAGAATTC | |  |  |
| pETSTS-R2 | CCTCGAGCTATGCAGAGTGATCAATTG | |  | *Xho* I |
| Fu28-F | GGACTAGTCGATCCCGCGAAATTAATACG | pET*4CL3*-*T*-*STS* | | *Kpn* I |
| Fu28-R | ***TTTGACCGATTCCAT***GGATCCGCGACCCAT |  |  | linker |
| FuSTS-F | ***GCTTGCGGCCGC***ATGGAATCGGTCAAAGAATT |  |  |  |
| FuSTS-R | CCTCGAGCTATGCAGAGTGATCAATTGCAACG |  |  | *Xho* I |
| pET4CL3-F2 | CCCGGTACCCCATGATTTCCGTAGCTAACAA |  |  | *Bam*H I |
| pET4CL3-R2 | CCTCGAG ACTAGTTTAGCTCAAAGGGGAAGCT |  |  | *Xho* I *Kpn* I |
| EGFP-F | GGGTACCATGGTGAGCAAGGGCGA | | pLGNL-*EGFP* | *Bam*H I |
| EGFP-R | GGGATCCCTTGTACAGCTCGTCCATGC | |  | *Spe* I |
| Ma4CL3-F3 | GGGATCCATGATTTCCGTAGCTAACAA | pLGNL-*EGFP::Ma4CL3* | | *Spe* I |
| Ma4CL3-R3 | GACTAGTCTACTCGAGGCTCAAAGGGG |  |  | *Kpn* I |
| Ma4CL3-F4 | GGAATTCATGATTTCCGTAGCTAACAA | pLGNL-*Ma4CL3::EGFP* | | *Eco*R I |
| Ma4CL3-R4 | GGGATCCCTACTCGAGGCTCAAAGGGG |  |  | *Spe* I |

*^a^* Short underlined nucleotides indicate restriction enzyme sites and bold italics nucleotides represent homology arms.
