# Supplementary material for: Correction: Characterization and Functional Analysis of 4-Coumarate:CoA Ligase Genes in Mulberry
Source: PLoS One. 2016 Jun 8;11(6):e0157414. doi: 10.1371/journal.pone.0157414 (PMC4898743; doi:10.1371/journal.pone.0157414)
Supplement: S1 File — (PDF) [file pone.0157414.s001.PDF]

RESEARCH ARTICLE

Characterization and Functional Analysis of 4-Coumarate:CoA Ligase Genes in Mul-berry

Chuan-Hong Wang, Jian Yu, Yu-Xiang Cai, Pan-Pan Zhu, Chang-Ying Liu, Ai-Chun Zhao, Rui-Hua Lü, Meng-Jiao Li, Feng-Xiang Xu, Mao-De Yu*

State Key Laboratory of Silkworm Genome Biology/College of Biotechnology, Southwest University, Chongqing, 400716, China

* yumd@163.com

Abstract

A small, multigene family encodes 4-coumarate:CoA ligases (4CLs) that catalyze the ligation of CoA to hydroxycinnamic acids, a branch point directing metabolites to flavonoid or monolignol pathways. In this study, we characterized four 4CL genes from *M*. *notabilis* Genome Database, and cloned four *Ma4CL* genes from *M*. *atropurpurea cv*. *Jialing* No.40. A tissue-specific expression analysis indicated that *Ma4CL3* was expressed at higher levels than the other genes, and that *Ma4CL3* was strongly expressed in root bark, stem bark, and old leaves. Additionally, the expression pattern of *Ma4CL3* was similar to the trend of the total flavonoid content throughout fruit development. A phylogenetic analysis suggested that Mn4CL1, Mn4CL2, and Mn4CL4 belong to class I 4CLs, and Mn4CL3 belongs to class II 4CLs. *Ma4CL* genes responded differently to a series of stresses. *Ma4CL3* expression was higher than that of the other *Ma4CL* genes following wounding, salicylic acid, and ultraviolet treatments. An in vitro enzyme assay indicated that 4-coumarate acid was the best substrate among cinnamic acid, 4-coumarate acid, and caffeate acid, but no catalytic activity to sinapate acid and ferulate acid. The results of subcellular localization experiments showed that Ma4CL3 localized to the cytomembrane, where it activated transcription. We used different vectors and strategies to fuse Ma4CL3 with stilbene synthase (STS) to construct four *Ma4CL-MaSTS* co-expression systems to generate resveratrol. The results indicated that only a transcriptional fusion vector, pET-*Ma4CL3-T-MaSTS*, which utilized a T7 promoter and *lac* operator for the expression of *MaSTS*, could synthesize resveratrol.

OPEN ACCESS

Citation: Wang C-H, Yu J, Cai Y-X, Zhu P-P, Liu C-Y, Zhao A-C, et al. (2016) Characterization and Functional Analysis of 4-Coumarate:CoA Ligase Genes in Mul-berry. PLoS ONE 11(5): e0155814. doi:10.1371/journal.pone.0155814

Editor: Hong Luo, Clemson University, UNITED STATES

Received: January 27, 2016

Accepted: May 4, 2016

Published: May 23, 2016

Copyright: © 2016 Wang et al. This is an open access article distributed under the terms of the Creative Commons Attribution License, which permits unrestricted use, distribution, and reproduction in any medium, provided the original author and source are credited.

Data Availability Statement: All relevant data are within the paper and its Supporting Information files.

Funding: Funded by Fundamental Research Funds for the Central Universities (grant No. XDJK2016D024), the China Special Fund for Agro-scientific Research in the Public Interest (grant No. 201403064), and the China Agriculture Research System (grant No.CARS-22).

Competing Interests: The authors have declared that no competing interests exist.

Introduction

The flow of carbon from primary metabolism for the biosynthesis of an array of phenylpropanoid secondary products involves a minimum of three enzymatic steps, which are catalyzed by phenylalanine ammonia-lyase (PAL), cinnamic 4-hydroxylase (C4H), and 4-coumarate:CoA ligase (4CL: EC 6.2.1.12). These enzymes collectively form the general phenylpropanoid pathway to maintain a continuous metabolic flux for the biosynthesis of phenylpropanoids [1,2] (Fig 1). 4CL is the last pivotal enzyme that catalyzes the production of 4-cinnamic acids or their derivatives into corresponding CoA esters to generate precursors that are needed for the

formation of a number of natural products, such as flavonoids, stilbenes, and lignin, which serve diverse functions, such as phytoalexins that protect against fungal infections, ultraviolet (UV) protectants, flower and fruit pigments, and structural components of cell walls [3–5].

The *4CL* genes exist in plants as a family with multiple members, including *Arabidopsis* (four members, [6]), *Oryza sativa* (five members, [7]) *Populus trichocarpa* (four members, [8]), and *Physcomitrella patens* (four members, [9]). However, 4CLs can be classified into two distinct groups in numerous plants: class I and class II. Class I has more relevance for lignin production, while class II 4CLs are more likely to biosynthesize flavonoids [6,10,11]. Previous studies that focused on lignin synthesis showed that the suppression of *4CL* expression may reduce lignin production in plants, such as *Nicotiana tabacum*, *Arabidopsis thaliana*, *Pinus radiata*, and *Populus tremuloides*, by 25 to 45% [10,12–14]. Additionally, resveratrol, one of the products of a branch of the phenylalanine metabolism pathway, has been proven to be beneficial to human health [15–17]. Furthermore, the co-expression of *4CL* with other genes to synthesize unnatural flavonoids, stilbenes, and other bioactive materials during fermentation has been investigated in recent studies [18,19].

Mulberry (*Morus alba* L.) leaves are the most ideal food for the domesticated silkworm (*Bombyx mori* L). Human utilization of the mulberry-silkworm interaction began at least 5,000

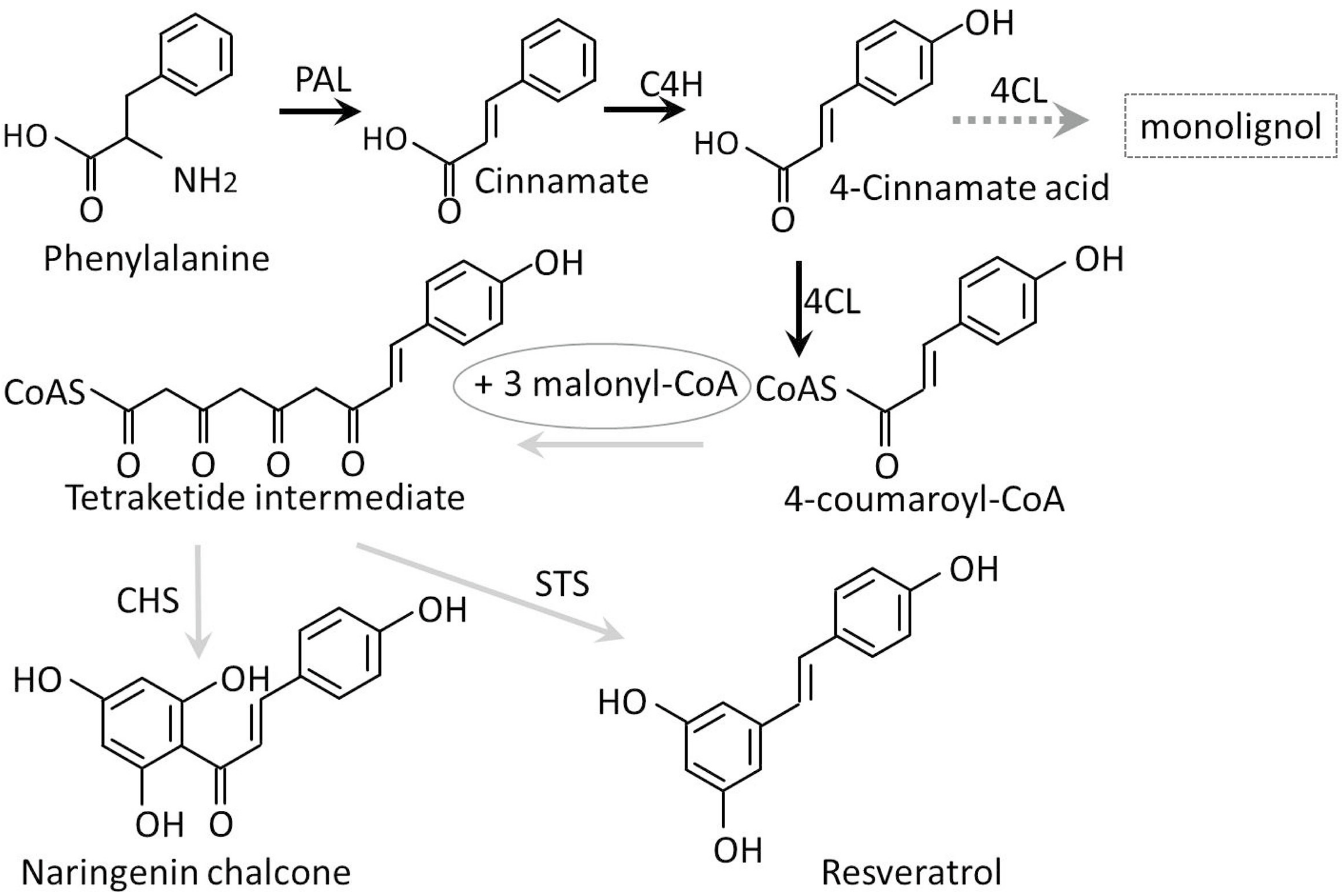

Fig 1. The enzymes of general phenylpropanoid metabolism, which are connected by black arrows, consist of PAL, C4H, and 4CL. Dashed arrows indicate a branch pathway for lignin biosynthesis emanating from the general phenylpropanoid metabolic pathway, which is also catalyzed by 4CL. The gray arrows indicate the pathways for chalcone and resveratrol biosynthesis.

doi:10.1371/journal.pone.0155814.g001

years ago and greatly influenced world history [20]. Furthermore, because of their rich secondary metabolite contents, different parts of the mulberry have been extensively investigated for their various health benefits. The mulberry fruit is widely regarded as a nutritious food, and it also has one of the highest anthocyanin contents of all fruits [21]. Moreover, mulberry leaves, root bark, and twigs have long been used in Chinese medicine [22]. Recent studies indicate that some mulberry phenylpropanoid metabolism products, such as anthocyanin, resveratrol, and mulberroside A, have antioxidative, antihyperglycemic, hypolipidemic, and antiatherogenic effects on human health [15–17,23]. Unfortunately, the genes in mulberry that are responsible for the synthesis of the aforementioned bioactive compounds are unknown. Thus, it is necessary to study the function of *Ma4CL* genes in mulberry. In this study, we report the isolation and characterization of four *Ma4CL* genes from mulberry, determine their tissue-specific expression, monitor their responses to wounding, UV, salicylic acid (SA), NaCl, and abscisic acid (ABA) stresses, and characterize the subcellular localization of Ma4CL3. Moreover, we evaluated different strategies to fuse *Ma4CL* genes with stilbene synthase (STS) to construct four *Ma4CL-MaSTS* co-expression systems to generate resveratrol.

Materials and Methods

Plant Materials and Multiple Stresses Test

The mulberry fruit materials were collected from the mulberry cultivar "*Jialing* No.40" at seven different developmental stages in the Southwest University mulberry garden. The 1-week-old etiolated seedlings for ABA (100μg/L) and NaCl (50mg/L) experiments were incubated at 25°C with 16-h light and 8-h dark photoperiods, and they were collected after stimulation for 1, 3, 6, 10, 16, and 24 h. Additionally, 10-week-old mulberry leaves were collected for UV-C (254 nm, Spectroline model ZW30S18Y, output 110 μW/cm^2 at 1 m), SA (100μg/L), and mechanical wounding treatments. In the SA treatments, a solution of SA was sprayed onto leaves, and mechanical wounding was performed by creating eight wounds along leaf veins using a sterile toothpick [24]. For the UV treatment, plants were irradiated by UV-C at a 15cm distance. All of the materials were frozen in liquid nitrogen and stored at −80°C.

Data Retrieval and Cloning of *Ma4CL* cDNAs

Similarity searches of the *Morus* Genome Database (http://morus.swu.edu.cn/morusdb/) were performed using the coding region of the *Pt4CL2* (*Populus tomentosa*, AFC89538.1), *Gh4CL1* (*Gossypium hirsutum* ACT32027.1), and *At4CL1* (*A. thaliana*, NP_175579.1) genes. Candidate genes were identified using the nucleotide Basic Local Alignment Search Tool (BLASTN) and the String Matching Algorithms Research Tool (SMART) (http://smart.embl-heidelberg.de/). Four *Ma4CL* genes were finally selected for further study. The primers (S1 Table) were used to isolate the *Ma4CL* genes in *M. atropurpurea cv. Jialing* No.40. The purified PCR products were confirmed by sequencing (S1 File).

Sequence Alignment and Phylogenetic Analysis

A multiple sequence alignment was performed using MEGA 5.0 software, and a phylogenetic tree was constructed using MUSCLE 3.6 software based on the maximum likelihood method. Other 4CL amino acid sequences for the phylogenetic reconstruction were retrieved from Genbank (http://www.ncbi.nlm.nih.gov), including *P. tomentosa* (Poptr4CL1-5 3A9U_A, AFC89538.1, AFC89539.1, AFC89540.1, AFC89541.1), *G. hirsutum* (Gh4CL1 ACT32027.1), *N. tabacum* (Ntab4CL2 O24146.1), *Pinus taeda* (Pinta4CL P41636.1), *A. thaliana* (At4CL1-4 NP_175579.1, NP_188761.1, NP_176686.1, NP_188760.3), *O. sativa Japonica* (Os4CL1-5

NP_001061353.1, NP_001047819.1, NP_001046069.1, NP_001058252.1, NP_001061935.1), *Lolium perenne* (Lp4CL1 AAF37732.1), *Lithospermum erythrorhizon* (Le4CL1-2 BAA08365.1, BAA08366.2), *Rubus idaeus* (Ri4CL1-3 AAF91310.1, AAF91309.1, AAF91308.1), and *Glycine max* (Gm4CL1-4 NP_001236418.1, NP_001236236.1, NP_001237270.1, P31687.2).

RNA Extraction, cDNA Synthesis, and Quantitative Reverse Transcription-Polymerase Chain Reaction (qRT-PCR)

Total RNA was extracted from root bark, stem bark, old leaves, and young leaves using the RNA Extraction Kit (TaKaRa, Shiga, Japan), and the total RNA of mulberry fruit was extracted using the RNA Extraction Kit TransZol Plant Kit (TransGen Biotech, Beijing, China). To digest genomic DNA, the RNA samples were treated with DNase I (TaKaRa), and 2 μg of purified RNA was used to synthesize cDNA with Moloney murine leukemia virus reverse transcriptase (Promega, Madison, WI, USA) as directed by the manufacturer. Seven-fold diluted cDNA was used in qRT-PCR. The primers were designed using the online tool of the GeneScript Company (Nanjing, China) (http://www.genscript.com.cn/index.html) (S2 Table). Reactions were prepared according to the manufacturer's instructions using SYBR® Premix Ex Taq™ II (TaKaRa) and 2 μL of diluted cDNA in 20 μL reactions. The qRT-PCRs were conducted in the StepOne Real-Time PCR System (Applied Biosystems, Foster City, CA, USA). The *MaACTIN3* (HQ163775.1) gene was used as an internal control to normalize the relative expression of target genes [25]. All data was analyzed using $2^{-\Delta\Delta Ct}$method.

Total Flavonoid Determination

The total flavonoid content assay was conducted using a previously described protocol [26]. Different development stages of fruit were collected and dried in a vacuum freeze drier (Thermo Fisher Scientific, Waltham, MA, USA). Each sample (0.5 g) was ground to a powder at low temperature, extracted three times with 10 mL of 75% (v/v) ethanol for 1 h, and the filtrate was evaporated to dryness and dissolved in 5 mL of 75% (v/v) ethanol. Subsequently, 0.5 mL of $NaNO_2$ (50 mg mL^{-1}) was added to 0.1 mL of the extract, shaken vigorously, and the mixture was left at room temperature for 6 min. Then, 0.5 mL of $Al(NO_3)_3$ was added to the reaction mixture. After 6 min incubation at room temperature, 2.5 mL of NaOH (1M) was added to the mixture and incubated at room temperature for 15 min. The absorbance was determined at 510 nm.

Subcellular Localization of the Ma4CL3 Protein

Ma4CL3 was fused with the 5' and 3' ends, respectively, of the enhanced green fluorescent protein (EGFP) gene to construct a fusion protein expression cassette. First, *EGFP* was inserted into the *Spe* I/*Bam* HI sites of the plant expression vector pLGNL to construct pLGNL-*EGFP*. Then, *Ma4CL3* was inserted into the *Bam*H I/*Kpn* I and *Eco*R I/*Spe* I sites to construct pLGNL-*Ma4CL3*::*EGFP* and pLGNL-*EGFP*::*Ma4CL3*, respectively. The gene-specific primers that contained the aforementioned restriction enzyme sites are shown in S3 Table. The vectors that contained *EGFP*, *Ma4CL3*::*EGFP* and *EGFP*::*Ma4CL3* fusion genes were confirmed by sequencing (Sangon Biotech, Shanghai, China) and then they were transiently introduced into onion (*Allium cepa* L.) epidermal cells by *Agrobacterium tumefaciens* strain LBA4404. The onion epidermis was stained with 4', 6-diamidino-2-phenylindole (DAPI) before preparing microscope slides, and images were captured with a fluorescence microscope (Olympus FV1000, Tokyo, Japan).

Recombinant Protein Expression and Purification

Full-length *Ma4CL3* cDNA was cloned into the pET28a(+) vector between the *Hin*d III and *Xho* I sites (S3 Table) to obtain fusion proteins containing an amino-terminal hexahistidine tag. The resulting plasmids were transformed into *Escherichia coli* BL21 (DE3) pLysS cells. Cultures were grown until the optical density at 600 nm (OD_{600}) reached approximately 0.6, at which point they were induced with 0.1 mM isopropyl-β-D-thiogalactopyranoside (IPTG) for 12 h at 16°C, and then the cells were collected by centrifugation. Recombinant enzymes were purified by a Ni-NTA metal-affinity matrix (GenScript) according to the instructions of the manufacturer. Then, protein bands in the lanes of a 12% polyacrylamide gel were identified using a UltraFlex III matrix-assisted laser desorption ionization-tandem time of flight mass spectrometer (MALDI-TOF/TOF MS) (S2 File). The peptide fragment ion data acquired from the MALDI-TOF/TOF MS were used to search for protein candidates in the *Morus* Genome Database (http://morus.swu.edu.cn/morusdb/).

Enzyme Assay

Enzyme assays were conducted as described previously [6], with a few modifications. Reactions consisted of 2 mL of Tris-HCl (100 mM) containing 5 μg of affinity-purified protein, 2.5 mM $MgCl_2$, 2.5 mM ATP, and a range of cinnamic acid, 4-coumarate acid, caffeate acid, sinapate acid and ferulate acid; the reaction was started by the addition of 0.2 mM CoA. The absorbance was measured at 311, 333, 345, 346, and 352 nm for the corresponding cinnamoyl-CoA, 4-coumaroyl-CoA, feruloyl-CoA, caffeoyl-CoA, and sinapoyl-CoA products, respectively, against a blank group. The extinction coefficients (ε) were 22, 21, 19, 18, and 20 mM^{-1} cm^{-1}, respectively, for the corresponding CoA ester complexes.

Fermentation Tests

To study other characteristics of *Ma4CL3*, several strategies were used to co-express it with *MaSTS* (GenBank: ALS20364.1). All primers used for generating fusion proteins are listed in S3 Table, and the fusion strategy is described in Fig 2.

4-S. The *Ma4CL3* gene was amplified with primers pETDuet*4CL3*-F (containing a *Pst* I site) and pETDuet*4CL3*-R (containing a *Hin*d III site) and cloned into the same sites of the pETDuet-1 plasmid, which resulted in the pETDuet-*4CL3* vector. The *MaSTS* gene was amplified by PCR using primers *MaSTS*-F (*Kpn* I) and *MaSTS*-R (*Xho* I). The *MaSTS* gene was digested and inserted into the *Xho* I/*Kpn* I sites of the pETDuet-*4CL3* vector, resulting in pETDuet-*4CL3-STS*. Plasmid pACYCDuet-*4CL3-STS* was assembled in the same manner as plasmid pACYCDuet-*4CL3-STS*.

S4 and 4S. As in a previous study [27], we also generated two different types of translational fusions between Ma4CL3 and MaSTS, which were carried by plasmid pET28a(+). The first was the fusion of Ma4CL3 to the amino-terminus of MaSTS, which resulted in pET*4CL3-STS* and referred to as 4S. The second was the fusion of MaSTS to the amino-terminus of Ma4CL3, which resulted in pET*STS-4CL3* and referred to as S4. The original stop codon of the first gene, which was closest to the promoter, was mutated to TCA (encoding Ser), and a mini-linker encoding Ser-Ser-Gly-Ser-Gly was inserted in front of the second gene.

4-T-S. *Ma4CL3* carried a *Bam*H I site at its 5'-end, and *Kpn* I and *Xho* I sites at its 3'-end, and it was first cloned into the pET28a(+) vector between the *Bam*H I and *Xho* I sites, which resulted in the pET*4CL3*. The pET28a(+) fragment from AGATCTCGATCCCGCGAA to GGATCC (the *Bam*H I site) was cloned and mutated from AGATCT to CTGCAG (a *Kpn* I site), and then it was linked to the 5'-end of *MaSTS* by fusion PCR. Then, the digested fusion fragment was inserted into the *Kpn* I/*Xho* I sites of pET*4CL3*, which resulted in pET*4CL3-T-STS*. Thus, the genes *Ma4CL3* and *MaSTS* were preceded by a T7 promoter/*lac* operator and a ribosome-binding site.

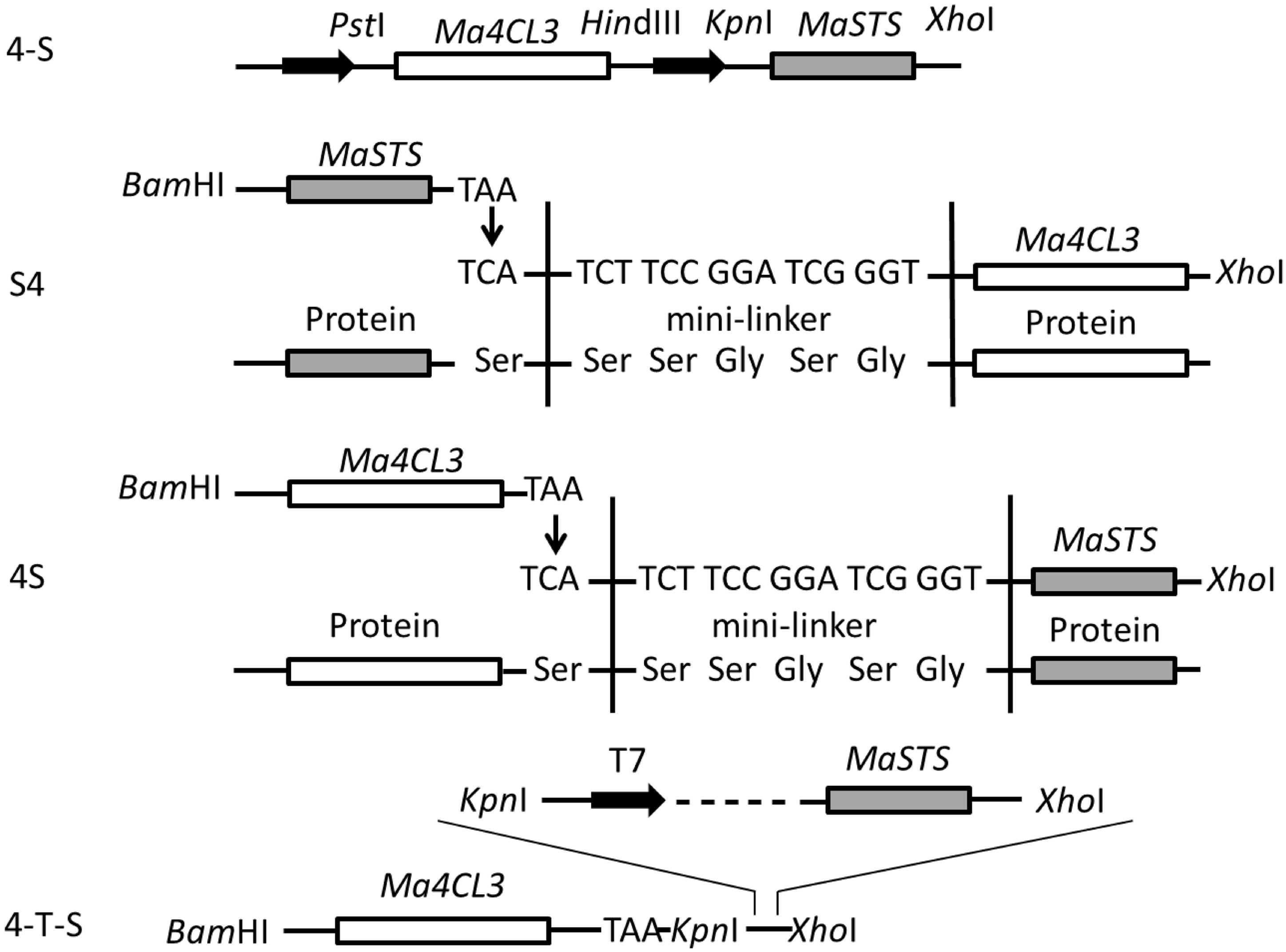

Fig 2. Schematic diagram representing the co-expression of the Ma4CL3 and MaSTS proteins. 4-S was carried by plasmid pETDuet-1/pACYCDuet-1; S4 and 4S were carried by plasmid pET28a(+); 4-T-S was also carried by plasmid pET28a(+), but with an independent T7 promoter and *lac* operator.

doi:10.1371/journal.pone.0155814.g002

The four aforementioned co-expression plasmids were transformed separately into *E. coli* BL21 (DE3) pLysS. The cells were incubated at 28°C until the OD_{600} reached 0.6, induced with 0.1 mM IPTG, and grown for 10 h at 16°C for protein production. Then, the cells were collected by centrifugation at 5,000 × *g* 5 min and resuspended in M9 medium containing 1 mM 4-coumarate acid, 0.1 mM malonyl-CoA, 0.1 mM IPTG, and necessary antibiotics. After incubation at 25°C for 24 h, lysed fermentation liquor was obtained by sonication, and the liquor was centrifuged at 12,000 × *g* for 20 min to pellet insoluble debris. Subsequently, it was extracted with ethyl acetate, and 1 mL organic phase samples were collected for high-pressure liquid chromatography (HPLC) analysis.

HPLC Analysis

A Waters 2487 HPLC system (Waters, Milford, MA, USA) and a reverse phase 5 μm C18 column (4.6×150 mm) (Waters Sunfire™, Dublin, Ireland) were used for the analyses. The injection volume was 10 μL, and the flow rate was 0.8 mL/min. The separation temperature was

25°C, and detection was at 306 nm. Ethyl acetate samples were centrifuged and filtered through a 0.22 μm filter. The mobile phases were (A) methyl alcohol and (B) 0.1% aqueous acetic acid. The solvent gradient elution program was as follows: 0–10 min, 10–30% A, 90–70% B; 10–25 min, 30–50% A, 70–50% B; 25–30 min, 50–70% A, 50–30% B; 30–45 min, 70–30% A, 30–70% B; 45–50 min, 30–10% A, 70–90% B.

Statistical Analysis

For all the qRT-PCR experiments, triplicate samples were performed, and the results were expressed as mean ± standard deviation (SD). Statistical analysis was performed using SPSS Statistics 18.0 (SPSS Inc., Chicago, IL, USA) with Duncan's multiple range test and figures were drawn using by GraphPad Prism v5.0 software (GraphPad Software Inc., La Jolla, CA, USA). Mean values that were significantly different within treatment from each other were indicated by"*".

Results

Structure and Phylogenetic Analysis of 4CL Genes

Based on a multiple sequence alignment of sequences obtained from the *Morus* Genome Database and GenBank, four *Mn4CL* genes were found, and they were confirmed by isolating their corresponding cDNAs from various tissues. Like *4CL* genes in other species, the *Mn4CL* genes have a multi-exon and -intron structure. The exon and intron structures for the *Mn4CL* genes are shown in Fig 3. *Mn4CL1* and *Mn4CL3* contain six exons and five introns, and *Mn4CL2* contains five exons and four introns. However, *Mn4CL4* only contains two exons and one intron. All of the *Mn4CL* genes were predicted to encode a family of proteins that includes Mn4CL1 with 646 amino acids, Mn4CL2 with 546 amino acids, Mn4CL3 with 595 amino acids, and Mn4CL4 with 407 amino acids (S3 File). The results of the phylogenetic analysis obviously divided the sequences into two groups, suggesting that Mn4CL3 belongs to the class II group; whereas Mn4CL1, Mn4CL2, and Mn4CL4 belong to the class I group (Fig 4). And the multiple sequence alignment indicated that the catalytic domains and active sites are conserved in these mulberry proteins, and that all four Mn4CLs have two structural domains, a putative AMP-binding motif (box I) and a conserved box II domain (Fig 5).

Expression of *Ma4CL* Genes in Different Tissues and Dynamic Expression under Multiple Stresses

To further investigate how *Ma4CL* genes are expressed in various tissues, transcripts from the four *Ma4CL* genes were quantitatively measured by qRT-PCR using gene-specific primers (S2

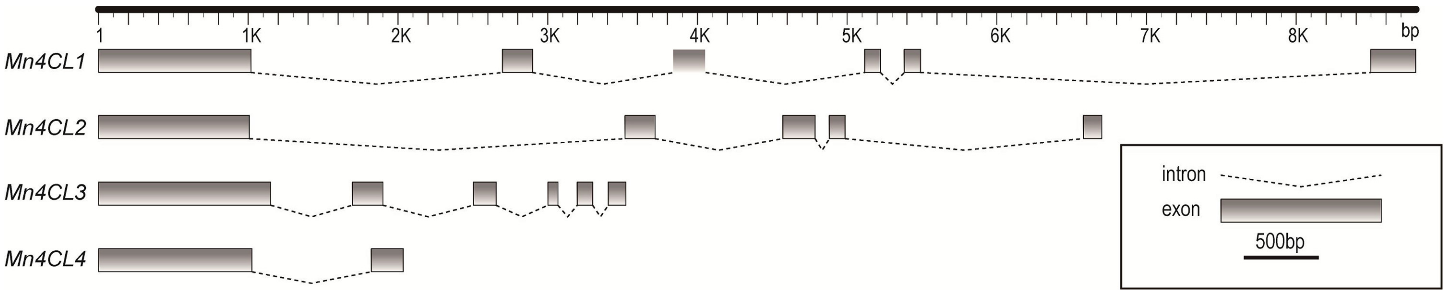

Fig 3. Gene structure of *Morus notabilis 4CL* genes. Schematic diagrams with exons (gray boxes) and introns (dashed lines between exons). Gene structures were displayed by Fancy Gene (http://bio.ieo.eu/fancygene/).

doi:10.1371/journal.pone.0155814.g003

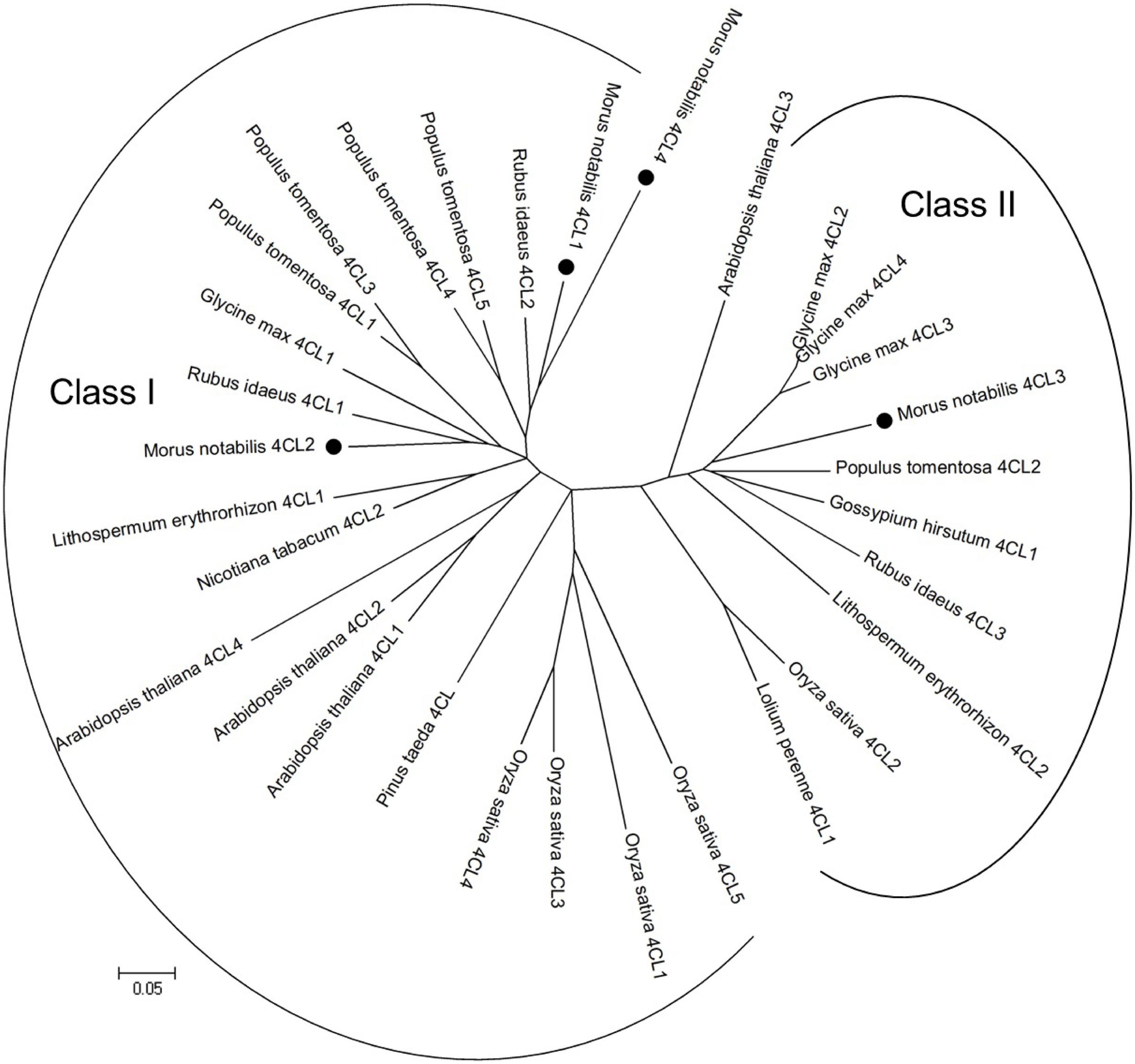

Fig 4. The phylogenetic tree analysis of Mn4CL proteins. The results of the phylogenetic analysis divided the sequences into two groups, suggesting that Mn4CL3 belongs to the class II group; whereas Mn4CL1, Mn4CL2, and Mn4CL4 belong to the class I group.

doi:10.1371/journal.pone.0155814.g004

Table). Total RNA was isolated from root bark, stem bark, old leaves, and young leaves of 10-week-old mulberry plants. As shown in Fig 6A, all of the *Ma4CL* genes were constitutively expressed in all selected organs, but they exhibited significant differences in their magnitudes of expression. The expression level of *Ma4CL3* was higher than those of the other three genes in the same tissues, and *Ma4CL3* was strongly expressed in root bark, stem bark, and old leaves.

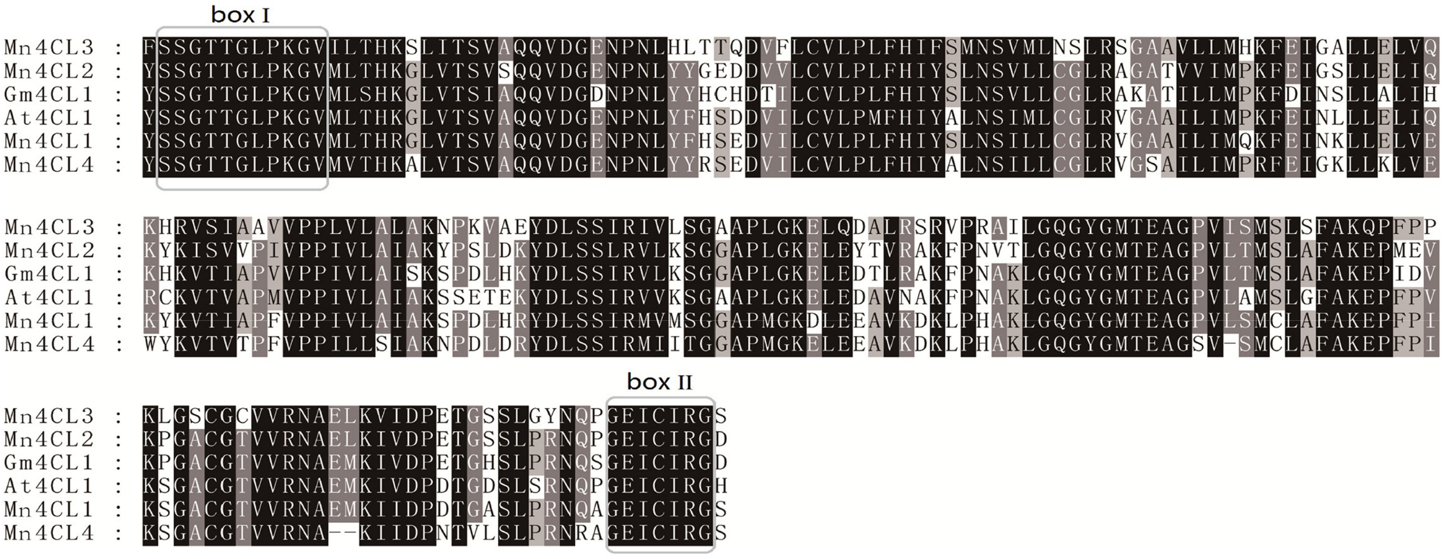

Fig 5. Sequence comparison of the deduced amino acid sequences. Box I represents the putative AMP-binding domain, and box II represents the conserved GEICIGR putative catalytic site.

doi:10.1371/journal.pone.0155814.g005

Furthermore, the *Ma4CL3* transcript was the most abundantly expressed transcript in old leaves, followed by root bark and stem bark, while the *Ma4CL3* transcript was the least abundant in young leaves. Additionally, *Ma4CL1* and *Ma4CL2* were highly expressed in root bark, while *Ma4CL4* exhibited the lowest expression in all selected organs.

The expression of both classes of *4CL* genes was induced by wounding (Fig 6B). *Ma4CL1* and *Ma4CL3* expression exhibited an "M-shaped" pattern, peaking at 1 and 24 h after leaves were wounded. *Ma4CL3* especially showed clear induction at 24 h, and the second expression peak was more than 4.11-fold greater than that of the control group. However, *Ma4CL2* and *Ma4CL4* were less responsive to wounding treatment than *Ma4CL1* and *Ma4CL3*, and their transcript levels peaked at 1 and 3 h, respectively.

The *Ma4CL* genes exhibited various responses to SA treatment (Fig 6C). *Ma4CL3* expression was sensitive to SA, peaked at 1 h, decreased to its normal level at 6 h, and sharply increased after 10 h of treatment, at which time its expression was approximately 7.06-fold greater than that of the control group. *Ma4CL1*, *Ma4CL2*, and *Ma4CL4* exhibited similar expression patterns, as evidenced by small increases in expression after SA treatment, followed by a return to their control levels.

The expression of the *Ma4CL* genes was UV-inducible (Fig 6D). Except for *Ma4CL4*, whose expression exhibited a single peak at 6 h, the expression of the other *Ma4CL* genes exhibited similar double-peaked patterns, and decreased at 10 h. However, *Ma4CL1* expression peaked at 3 and 16 h, while *Ma4CL2* expression peaked at 6 and 16 h. As seen for the other treatments, *Ma4CL3* expression was induced substantially by UV light, and it reached its maximum at 1 h, which was approximately 1.81-fold greater than that of the control group.

Expression of *Ma4CL* Genes and Total Flavonoid Determination during Mulberry Fruit Development

During mulberry fruit development from stage S1 to S7 (Fig 7E), four *Ma4CL* genes were constantly expressed throughout fruit development, but they showed two distinct expression patterns (Fig 7A–7D). *Ma4CL1*, *Ma4CL2*, and *Ma4CL4* were expressed at relatively high levels

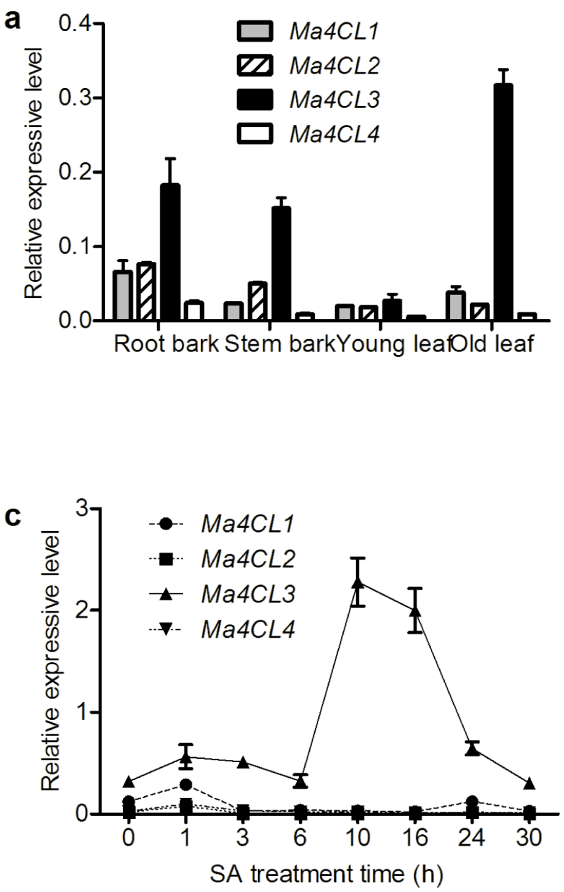

Fig 6. Expression of *Ma4CL* genes in different tissues and different response patterns under diverse stresses. All of the *Ma4CL* genes were constitutively expressed in all of the selected organs (a), but exhibited significant differences in their magnitudes of expression. *Ma4CL3* was expressed at higher levels than the other genes in the same tissues, and *Ma4CL3* was strongly expressed in root bark, stem bark, and old leaves. The *Ma4CL* genes showed different response patterns under diverse stresses, as the induction of *Ma4CL3* expression was greater that of the other genes after wounding (b), SA (c), and UV (d) treatments. Error bars on each column indicate SDs from three replicates.

doi:10.1371/journal.pone.0155814.g006

during stage S1. Then, their expression dramatically decreased during S2, increased gradually, reached their maximum levels during stages S6, S3, and S3, respectively, and then decreased during subsequent fruit development. However, the *Ma4CL3* expression pattern was quite different from that of the others; its transcription level decreased before stage S4, and then increased rapidly, peaking at stage S5, and then declined slowly (Fig 7C). Dynamic total flavonoid contents during mulberry fruit development from stage S1 to S7 were determined. As shown in Fig 7E, the trend of the total flavonoid content was similar to that of the expression pattern of *Ma4CL3* throughout fruit development, although it peaked during stage S6.

Various up-regulated expression patterns occurred under diverse stresses. Under NaCl stress (Fig 8), *Ma4CL1* and *Ma4CL3* expression in stems peaked at 1 h, and then decreased (Fig a and c). *Ma4CL2* and *Ma4CL4* exhibited similar double-peaked expression patterns (Fig 8B and 8D). In roots, *Ma4CL1* and *Ma4CL2* expression peaked at 6 and 3 h, respectively (Fig 8E and 8F). However, the expression of *Ma4CL3* and *Ma4CL4* did not noticeably increase, and was obviously down-regulated at 1 and 12 h (Fig 8G and 8H).

For the ABA treatment, stems and roots showed different responses (Fig 9). When stems were subjected to ABA treatment, *Ma4CL1* and *Ma4CL2* expression peaked at 1 and 12 h,

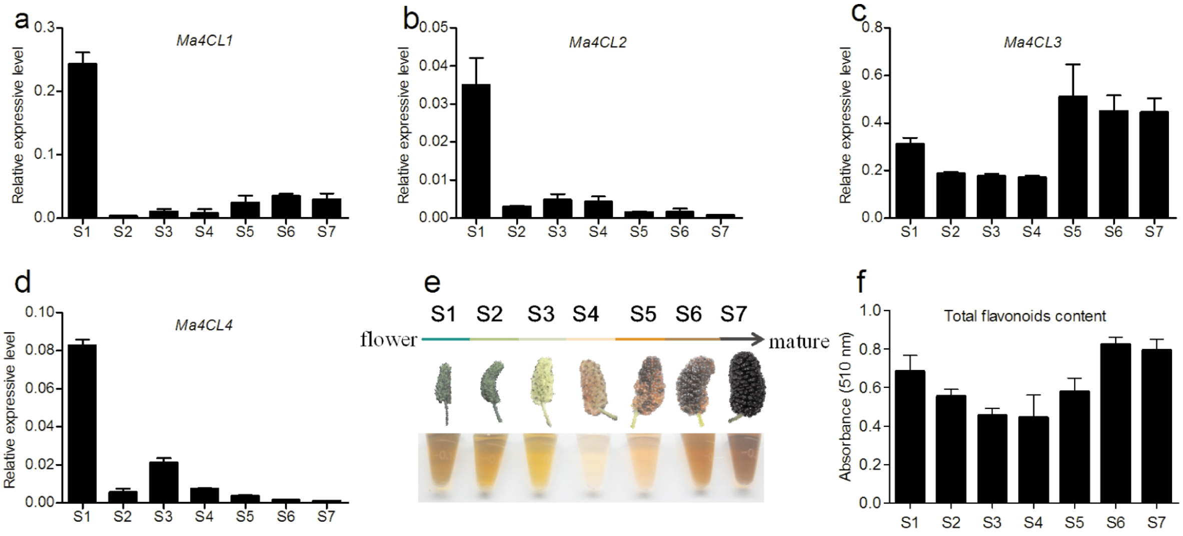

Fig 7. Expression of *Ma4CL* genes and total flavonoid contents during mulberry fruit development. a, b, c, and d represent *Ma4CL1*, *Ma4CL2*, *Ma4CL3*, and *Ma4CL4* expression levels, respectively, e and f indicate total flavonoid contents during fruit development from stage S1 to S7. Error bars on each column indicate SDs from three replicates.

doi:10.1371/journal.pone.0155814.g007

respectively, and then decreased (Fig 9A and 9B), while the expression levels of *Ma4CL3* and *Ma4CL4* did not change significantly (Fig 9C and 9D). However, the expression of *Ma4CL* genes in roots showed completely different responses. Except for *Ma4CL3*, whose expression reached its maximum after 3 h (Fig 9G), the expression levels of the other genes increased gradually and reached their maxima after 24 h of treatment (Fig 9E, 9F and 9H). Additionally, the ratio of expression level in roots and stems (RERS) also showed differences (Table 1). It can be obviously seen in Table 1 that the RERS of *Ma4CL2* were less than those of the control before 24 h, but the RERS of *Ma4CL3* and *Ma4CL4* were greater than those of the control.

After 4 d of ABA treatment, although all of the RERS were significantly up-regulated, *Ma4CL3* expression only increased 3.46-fold, while *Ma4CL1*, *Ma4CL2*, and *Ma4CL4* were up-

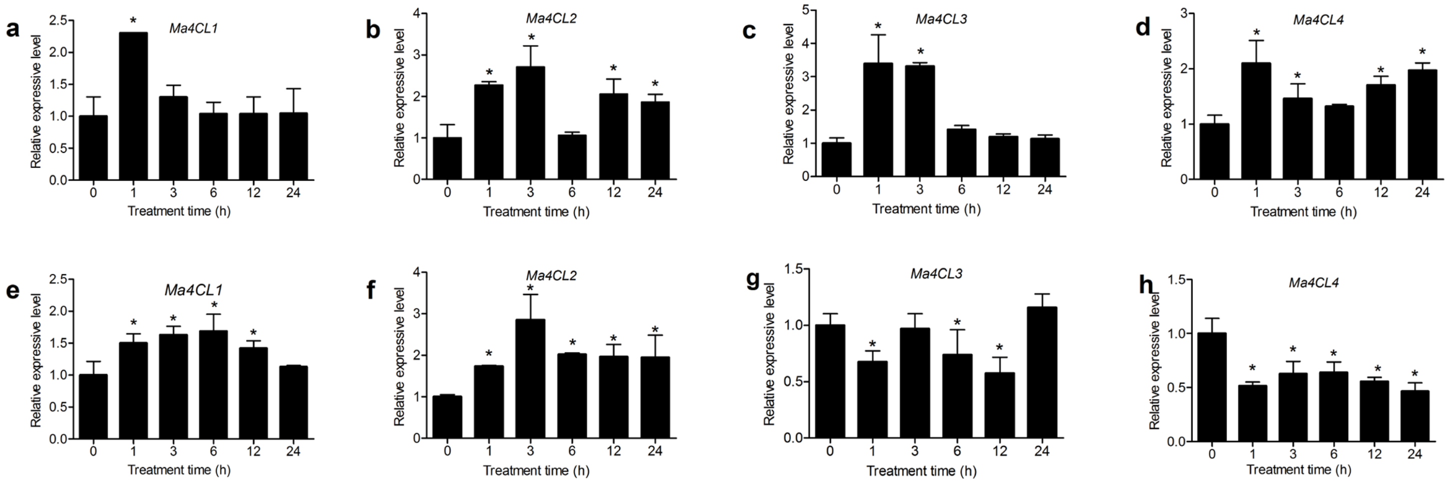

Fig 8. The relative expresa sion levels of *Ma4CL* genes under NaCl treatment. *Ma4CL1* and *Ma4CL3* expression in stems peaked at 1 h, *Ma4CL2* and *Ma4CL4* exhibited similar double-peaked expression patterns (a–d). In roots, *Ma4CL1* and *Ma4CL2* expression peaked at 6 and 3 h, respectively. However, the expression of *Ma4CL3* and *Ma4CL4* was down-regulated at 1 and 12 h (e–h). Error bars on each column indicate SDs from three replicates. Significant differences ($p < 0.05$) among treatment are marked with "*".

doi:10.1371/journal.pone.0155814.g008

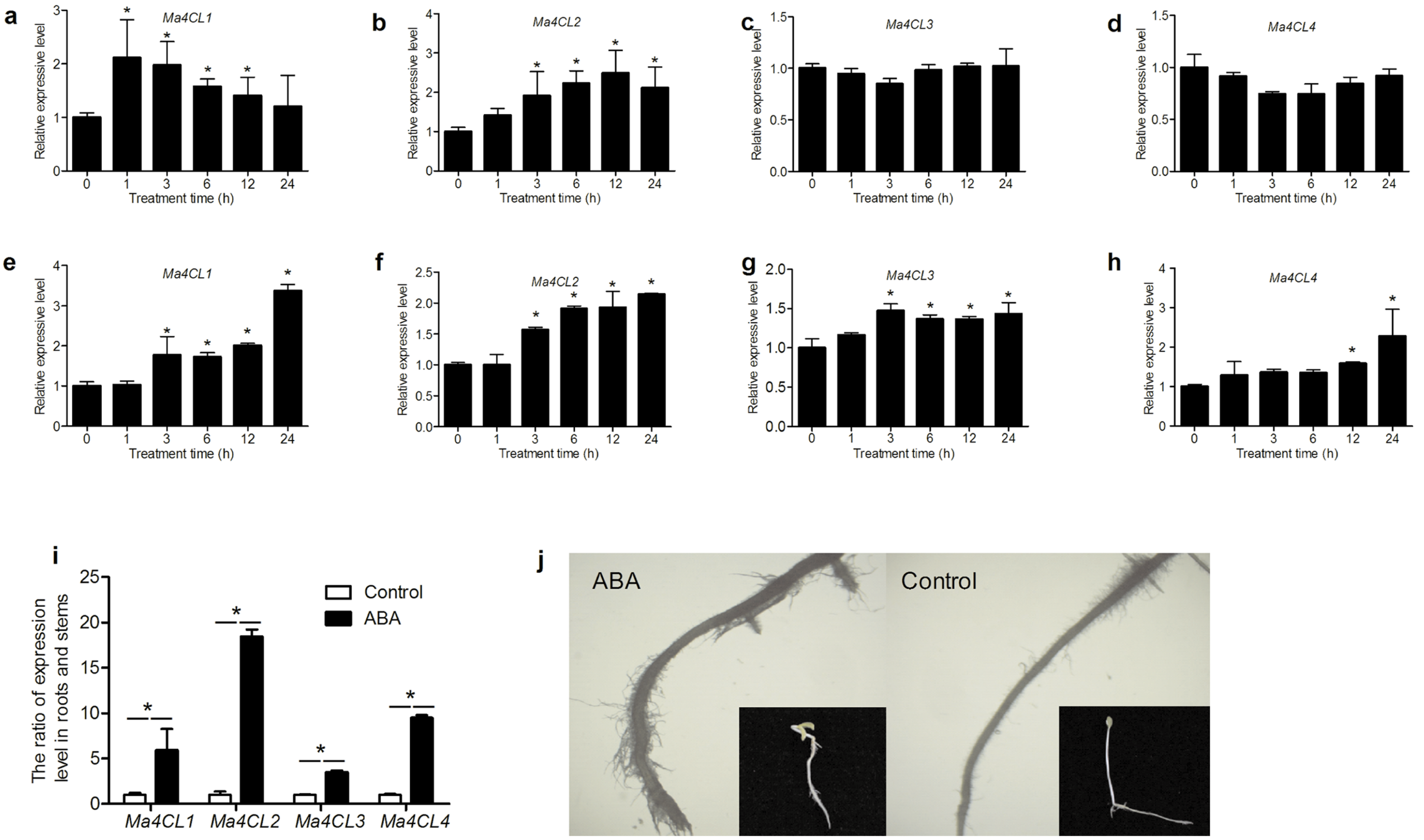

Fig 9. Relative expression levels of *Ma4CL* gene under ABA treatment. *Ma4CL* genes in stems (a–d) and roots (e–h) showed different responses. *Ma4CL1* and *Ma4CL2* expression in stems peaked at 1 and 12 h, respectively, while *Ma4CL3* and *Ma4CL4* expression did not change significantly. However, *Ma4CL3* expression in roots reached its maximum after 3 h, while the expression levels of the other genes increased gradually and reached their maxima after 24 h of treatment. Significant differences ($p < 0.05$) among treatment are marked with "*". After 4 d of ABA treatment (i), although all of the RERS were significantly up-regulated, *Ma4CL3* expression was only up-regulated 3.46-fold, while *Ma4CL1*, *Ma4CL2*, and *Ma4CL4* expression was up-regulated approximately 5.23-, 16.50-, and 9.47-, respectively, compared with that of the control ("*" indicates $p < 0.01$). (j) The ratio of root length and stem length changed significantly under ABA treatment, and taproots became sturdier than control, and the number of root hairs increased significantly. Error bars on each column indicate SDs from three replicates.

doi:10.1371/journal.pone.0155814.g009

regulated approximately 5.23-, 16.50-, and 9.47-fold, respectively, compared with that of the control group (Fig 9I). Furthermore, the ratio of root length and stem length reached 3.49, while that of the control group was only 1.12. Additionally, the taproots of the ABA treatment

Table 1. The ratio of expression level in roots and stems under the ABA treatment.

Treatment time	*MaCL1*	*MaCL2*	*MaCL3*	*MaCL4*
0 h	1.249 ± 0.22	2.220 ± 0.03	0.554 ± 0.09	0.593 ± 0.01
1 h	0.600 ± 0.12*	1.573 ± 0.33*	0.683 ± 0.11	0.833 ± 0.03**
3 h	1.113 ± 0.33	1.817 ± 0.27	0.963 ± 0.07**	1.082 ± 0.09**
6 h	1.358 ± 0.07	1.902 ± 0.71	0.773 ± 0.15	1.079 ± 0.24**
12 h	1.765 ± 0.13**	1.717 ± 0.05*	0.743 ± 0.02	1.117 ± 0.16**
24 h	3.464 ± 0.87**	2.248 ± 0.11	0.778 ± 0.13	1.471 ± 0.29**

Column values without "*" indicate no significant difference in expression compared to 0 h (P > 0.05). One "*" indicates a significant down-regulation in expression compared with that at 0 h; a double "*" indicates a significant up-regulation in expression compared with that at 0 h. Results are displayed with means ± standard deviations (n = 3).

doi:10.1371/journal.pone.0155814.t001

group became sturdier than control, and the number of root hairs increased significantly (Fig 9J).

Subcellular Localization of the Ma4CL3 Protein

To study the localization of the Ma4CL3 protein, *EGFP*, *Ma4CL3*::*EGFP*, and the *EGFP*:: *Ma4CL3* fusion genes, under the control of the Cauliflower mosaic virus (CaMV) 35S promoter (Fig 10A), were transfected into onion epidermal cells. The *EGFP* vector was used as a

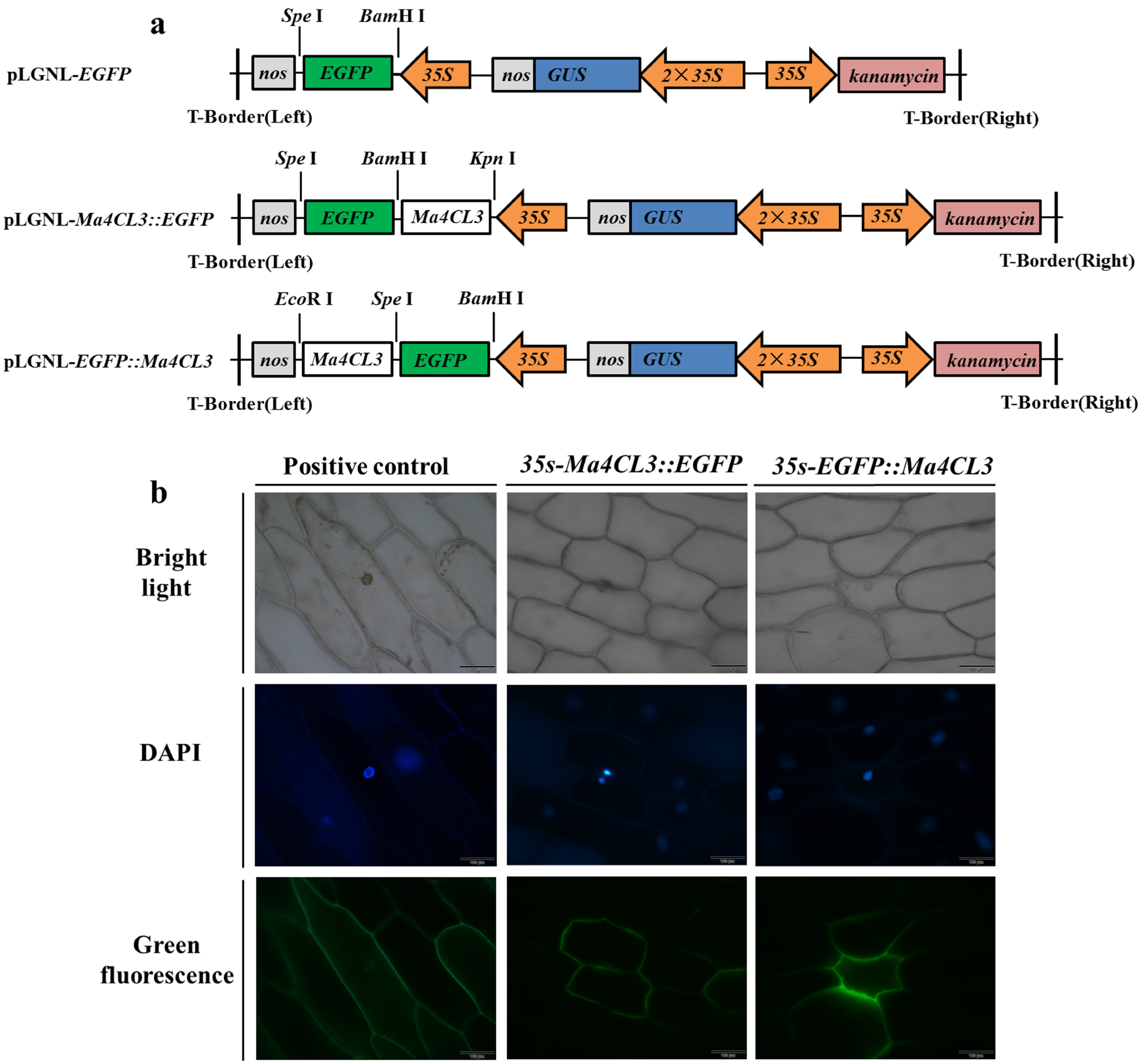

Fig 10. Subcellular localization of the Ma4CL3 protein. (a) *EGFP*, *Ma4CL3*::*EGFP*, and the *EGFP*::*Ma4CL3* fusion strategies. (b) The positive control was a transfected plasmid containing *EGFP* alone. DAPI was used to indicate the nucleus. It was observed that Ma4CL3 localized to the cytomembrane mainly.

doi:10.1371/journal.pone.0155814.g010

control and was transfected separately. It could be clearly observed that the green fluorescence signals of the EGFP distributed in the cytoplasm, nucleus and cytomembrane (Fig 10B). However, the green fluorescence signals of the Ma4CL3::EGFP and EGFP::Ma4CL3 fusion proteins were observed mainly at the cytomembrane, where they may activate transcription, which implied that Ma4CL3 is a cytomembrane-localized protein. This result is consistent with the protein function of the 4CL family.

Enzyme Assay of Ma4CL3

The open reading frame of *Ma4CL3* was subcloned into the expression vector pET28a(+), and soluble proteins were obtained. After purification via metal-affinity chromatography, the purified Ma4CL3 protein was analyzed by MALDI-TOF/TOF MS, and the results showed that the purified protein peptides completely matched the predicted peptides (S2 File). Enzyme assays of Ma4CL3 using various substrates, including cinnamic acid, 4-coumarate acid, caffeate acid, sinapate acid, and ferulate acid, were conducted. As shown in Table 2, the K_m of 4-coumarate acid was 10.49 μM; however, the $V_{\max}$ was only 4.4 nkat mg^{-1}. This indicated that 4-coumarate acid was the best substrate among cinnamic acid, 4-coumarate acid, and caffeate acid, while no catalytic activity was observed when sinapate acid and ferulate acid were used as substrates.

Fermentation Test

As described in the Methods section, four co-expression strategies were used. Unfortunately, only the 4-T-S strategy produced two soluble proteins in a low-temperature (16°C) and low-IPTG (0.1 mM) fermentation environment, but only 0.1268 mg/L resveratrol was produced in the fermentation broth (Fig 11). We were unable to obtain soluble Ma4CL3, Ma4CL3-MaSTS, or MaSTS-Ma4CL3 proteins using the 4-S, 4S, and S4 strategies.

Discussion

The phenylalanine metabolic pathway, as one of the most important carbon flux metabolic pathways, plays an important role in carbon accumulation during plant growth. 4CL is the key enzyme in the phenylalanine metabolic pathway, and it is regulated developmentally and activated by external stimuli, such as pathogen infections, elicitor treatments, wounding, and UV light irradiation [2,4]. Additionally, it shows different catalytic activity for various cinnamate derivative substrates, such as cinnamic acid, 4-coumarate acid, caffeate acid, sinapate acid and ferulate acid, and it has a defined substrate preference [9,28–30]. The preference is beneficial for metabolism, as it can provide downstream processes with suitable proportions of cinnamoyl CoA mixtures, and it regulates the metabolites flowing to lignin or non-lignin syntheses.

Similar to *4CL* genes in other species, *Mn4CL* genes have multiple introns and exons, which differ from each other, both in terms of their lengths and nucleic acid sequences. However, box I and box II regions are conserved peptide sequences in the Mn4CL family. Box I (SSGTTGLPKGV) is the AMP-binding domain, and box II (GEICVRS) is the catalytic site [29]

Table 2. Kinetic characterizzation of Ma4CL3.

Isoenzyme Substrate	K_m (μM)	$V_{\max}$ (nKat mg^{-1})	K_{cat} (s^{-1})	K_{cat} / K_m (μM^{-1} s^{-1})
Cinnamic acid	73.53	22.15	1.041	0.014
4-Coumarate acid	10.49	4.40	0.284	0.027
Caffeate acid	463.69	16.15	1.428	0.003

Ma4CL3 was no catalytic activity to Sinapate acid and ferulate acid.

doi:10.1371/journal.pone.0155814.t002

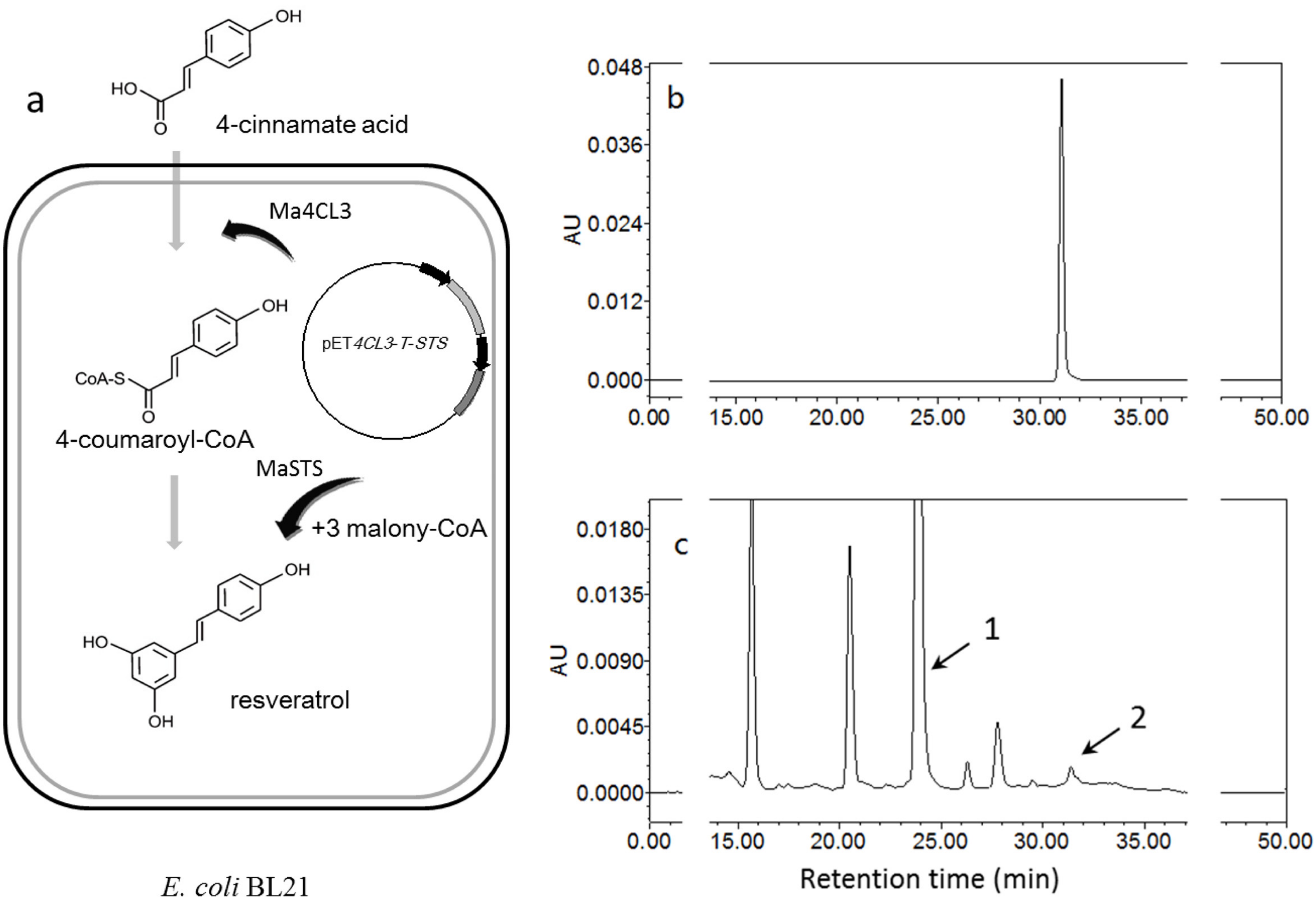

Fig 11. Fermentation test and HPLC detected. (a) Biosynthesis routes of resveratrol constructed in recombinant *E. coli*.. Standard chromatograms of resveratrol (b) and the ethyl acetate extract (c). Peak 1 represents 4-cinnamate acid, and peak 2 represents resveratrol.

doi:10.1371/journal.pone.0155814.g011

(Fig 5). During different periods of mulberry fruit growth, the expression level of *Ma4CL3* was higher than that of the other three genes, and the expression trend of *Ma4CL3* was in accordance with the trend of the total flavonoid content in fruits. The maximum expression of *Ma4CL3* did not occur in the S7 stage when the color was the deepest, but during the S5 stage. It was during the S5 stage that the flavonoid content also began to increase. This is similar to the expression pattern of *Nt4CL2* (*N. tabacum*) in tobacco flowers, which exhibit high expression in unpigmented corolla tubes, and high *Nt4CL2* transcript levels are first evident in stage-4 flowers, and decrease significantly in stage-6 flowers [2]. In addition, according to the mulberry tissue-specific expression analysis, *Ma4CL3* transcripts are mainly present in the root bark, stem bark, and old leaves. Moreover, root bark, stem bark, and old leaves are the regions that are enriched in flavonoids [26,31]. It was found that recombinant Ma4CL3 has greater affinity for 4-coumarate acid than other substrates in the in vitro enzyme activity test, and the product of 4-cinnamoyl-CoA was the most-used precursor for flavonoid biosynthesis. Previous studies indicated that the K_m of 4CL for flavonoids was usually lower than that for monolignols [6,9,28,30,32]. Furthermore, a phylogenetic tree suggested that Mn4CL3 belonged to class II. In various plant species, the biosynthesis of monolignols has been considered to be the main

function of class I 4CL, and the main function of class II 4CL has been suggested to be associated with flavonoid synthesis [6,10,11]. A recent study proved that a mutation in *At4CL3* (*A. thaliana*) significantly reduced the flavonol glycoside content in *Arabidopsis* by approximate 80% compared with that of wild-type plants, while mutations in the other *At4CL* (class I) genes did not significantly affect the flavonol glycoside content [33]. Additionally, silencing of both the *At4CL1* and *At4CL2* (class I) genes reduced the amounts of total lignin compared with that of non-induced stems [34]. Furthermore, suppression of *Os4CL3* (class I) expression resulted in a significant reduction in lignin [7]. In view of the above experiments, we infer that the primary function of Ma4CL3 is in flavonoid biosynthesis.

The synthesis of secondary metabolites in response to stress conditions, such as fungal, UV, and H_2O_2 treatments, has been implicated as a major defense response of higher plants [3,4,35]. Because flavonoids strongly absorb UV irradiation, the induced synthesis of flavonoids can afford plants protection against UV light [4]. Cell suspension cultures of parsley (*Petroselinum hortense*) have been studied extensively as a model system, and they have been shown to respond to UV irradiation by synthesizing flavonoids. Studies have shown that UV irradiation up-regulates the expression of several genes, including *PAL*, *4CL*, and *CHS*, which are essential genes for flavonoid synthesis [3,35]. When plants suffer from mechanical wounding, the cell walls of the wound site are first strengthened by crosslinking proteins to prevent dehydration and possible pathogen infections, and phenylpropanoid derivatives are required for lignification and suberization of the cell walls in a subsequent step [36]. In *A. thaliana*, *At4CL1* and *At4CL2* (class I) transcripts rapidly accumulated after wounding, but *At4CL3* (class II) expression was not wound responsive [6]. These results are in contrast to this study, in which wounding significantly induced the expression of *Ma4CL3* in leaves; however, *Ma4CL2* and *Ma4CL4* expression was less responsive to wounding treatment. This indicated that *Ma4CL3* was more sensitive to wounding treatment. Similar wounding of silver birch (*Betula pendula*) induced the expression of *Bp4CL1* (class I) and *Bp4CL2* (class II) genes in leaves, whereas the transcript levels of *Bp4CL3* and *Bp4CL4* (class I) remained unchanged [37]. Previous study showed that wounding can induce the expression of genes that encode enzymes of the general phenylpropanoid route, thereby leading to the generation of monolignols that are essential for wound sealing [38]. Therefore, the phenylpropanoid flux should be directed from the flavonoid biosynthesis route towards the biosynthesis of monolignols. Furthermore, wounding has been shown to decrease the expression of potential lignin biosynthesis repressors [39,40], which may be why *Ma4CL3* expression was induced in response to wounding stress.

Mulberry trees are ecologically and economically important perennial woody plants, which can grow in the severe desertification region and can adapt to drought, sand damage, and saline environments [41]. As important signaling molecules in the plant immune response, ABA and SA have been reported to improve tolerances to disease, salt, and dehydration [42,43]. However, there are only a few reports of the effect of SA on 4CL regulation. The results of this study showed that the expression of four *Ma4CL* genes was induced by SA (Fig 6B). The expression level of *Ma4CL3* was significantly up-regulated (7.06-fold compared with that of the control group), while the expression of the other three genes was unchanged. The results indicated that *Ma4CL3* is the most important participant in the SA response. Drought and salt lead to increased reactive oxygen species concentrations in the cell, and they stimulate the synthesis and accumulation of ABA in plant roots [43]. This induces certain physiological and biochemical responses in plants, which enable them to survive in such environments. In this study, the expression of the *Ma4CL* genes differed in roots and stems under the ABA treatment, the RERS of *Ma4CL3* increased slowly, while those of *Ma4CL1*, *Ma4CL2*, and *Ma4CL4* increased rapidly. At the same time, the ratio of root length and stem length, as well as lateral roots increased significantly. Accordingly, this suggests that after ABA stimulation, mulberry

enhanced the development of its belowground portion, while suppressing the development of its aboveground portion, to resist environmental stresses. For example, this strengthens the ability of mulberry to absorb water that is deep below the surface, while reducing water evaporation from aboveground organs. The development of plant roots is also related to lignin and the increased RERS of *Ma4CL* genes. However, under NaCl and ABA stresses, the expression of *Ma4CL* genes differed. This implies that mulberry responds to salt stress via an ABA-independent signal transduction pathway.

During the course of plant evolution, the functions of genes in the same family may be redundant or different. In this study, it was found that the four *Ma4CL* genes reacted differently to external stimuli. Some were more sensitive to the external stimuli, while others were not, and some exhibited the same reaction mode, which means that these isoforms may have similar regulatory effects and function redundantly with each other. *4CL* genes have been detected by monitoring their transcript levels and/or by promoter analyses using *Arabidopsis* (*A. thaliana*) [6,38], tobacco (*N. tabacum*) [44], rice (*O. sativa*) [7], and aspen (*P. trichocarpa*) [10]. The results demonstrated that the regulation of *4CL* genes is complex, and that the genes play both positive and negative roles [38]; thus, different 4CL family members show distinct expression patterns in response to biotic or abiotic stress [6,7].

It is well know that soluble protein is a prerequisite for protein activity. In this study, soluble Ma4CL3 could not be obtained at a high concentration of IPTG or at a high temperature, despite using multiple expression vectors. Previous studies have used the 4CL-STS co-expression system to ferment resveratrol, and the resveratrol content in broth was much higher than in this study [18,45–48]. It should be noted that the efficacy of recombinant microorganisms for resveratrol production depends on various factors, such as the species and the strain, the origin of the transferred genes, as well as other parameters such as plasmids or precursors used [48]. For instance, resveratrol yield produced by BW27784 (*E. coli*) was 2.67 times the yield of *Saccharomyces cerevisiae* with the *Nt4CL* (*Nicotiana tabacum*) and *VivSTS* (*Vitis vinifera*) [45]. However, resveratrol yield of *At4CL* (*Arabidopsis thaliana*) and *AhSTS* (*Arachis hypogaea*) is more than 6.25 times compared with *Nt4CL* and *VivSTS* [45,46]. Meanwhile, the resveratrol yield of fusion protein *Le4CL* (*Lithospermum erythrohizon*) and *AhSTS* (*Arachis hypogaea*) reached 5.25 mg/L in yeast strain [47]. In this study, we explore the possibility of gaining resveratrol yielded by the fermentation of genes from mulberry. We will focus on optimizing the fermentation conditions to obtain higher resveratrol concentrations in future research.

With greater attention being paid to health issues, natural products have enjoyed great popularity. However, because of numerous byproducts of chemical synthetic drugs and the low content of natural products, environmental damage and pollution caused by the extensive use of raw materials has become increasingly serious [26]. Additionally, mulberry is rich in resveratrol, oxyresveratrol, and mulberroside A [31,49], which means that there is a highly efficient biological synthesis system in mulberry. This view is supported by the fact that we determined that the trend of *Ma4CL* expression is related to flavonoid synthesis, and we established a co-expression system to produce resveratrol in microbes, as an alternative to extracting resveratrol from plants or obtaining it via chemical synthesis. This study lays the foundation for future research on the phenylalanine metabolic pathway in mulberry.

Conclusion

In summary, four *Ma4CL* genes from mulberry were identified in our study. The *Ma4CL* genes were expressed in all selected organs, although significant differences in their expression were observed. In particular, *Ma4CL3* exhibited relatively high expression in root bark, stem bark, and old leaves. Based on an in vitro enzyme assay, expression in mulberry fruits, and its

response to multiple stresses, the function of Ma4CL3 may be associated with flavonoid synthesis, and Ma4CL3 localized to the cytomembrane. Furthermore, the co-expression of *Ma4CL3* and *MaSTS* was established, and resveratrol was successfully produced by fermentation. The results pave the way for future work on the phenylalanine metabolic pathway in mulberry.

Supporting Information

S1 File. The cDNA sequences of *Ma4CL* isolated from *M. atropurpurea cv. Jialing* No.40.
(DOCX)

S2 File. The Maldi date of Ma4CL3. The purified Ma4CL3 protein was analyzed by MALDI--TOF/TOF MS, and the results showed that the purified protein peptides completely matched the predicted peptides
(PDF)

S3 File. The predicted Mn4CLs protein sequences. All of the *Mn4CL* genes were predicted to encode a family of proteins that includes Mn4CL1 with 646 amino acids, Mn4CL2 with 546 amino acids, Mn4CL3 with 595 amino acids, and Mn4CL4 with 407 amino acids.
(DOCX)

S1 Table. List of primers used to isolate the *Ma4CL* genes in *M. atropurpurea cv. Jialing* No.40.
(DOCX)

S2 Table. Primers for qRT-PCR in this study.
(DOCX)

S3 Table. Oligonucleotides for vector construction in this study.
(DOCX)

Author Contributions

Conceived and designed the experiments: CW MY AZ. Performed the experiments: CW JY YC. Analyzed the data: WC PZ CL RL. Contributed reagents/materials/analysis tools: AZ MY. Wrote the paper: CW AZ. Provided assistance with the preparation of mulberry seedlings: ML FX.

References

1. Eyles A, Bonello P, Ganley R, Mohammed C (2010) Induced resistance to pests and pathogens in trees. New Phytol. 185:893–908. doi: 10.1111/j.1469-8137.2009.03127.x PMID: 20015067
2. Lee D, Douglas CJ (1996). Two Divergent Members of a Tobacco 4-Coumarate: Coenzyme A Ligase (4CL) Gene Family. Plant Physiol. 112:193–205. PMID: 8819324
3. Beggs C and Wellman E (1985) Analysis of light-controlled anthocyanin formation in coleoptiles of Zea mays L.: the role of UV-B, blue, red and far-red light. Photochem Photobiol. 41:481–486.
4. Chappell J and Hahlbrock K (1984) Transcription of plant defence genes in response to UV light or fungal elicitor. Nature. 311:76–78.
5. Hahlbrock K and Scheel D (1989) Physiology and molecular biology of phenylpropanoid metabolism. Annu Rev Plant Physiol Plant Mol Biol. 40:347–369.
6. Ehlting J, Büttner D, Wang Q, Douglas CJ, Somssich IE, Kombrink E. (1999) Three 4-coumarate:Coenzyme A ligases in *Arabidopsis thaliana* represent two evolutionarily divergent classes in angiosperms. Plant J. 19: 9–20. PMID: 10417722
7. Gui J, Shen J, Li L (2011) Functional Characterization of Evolutionarily Divergent 4-Coumarate:Coenzyme A Ligases in Rice. Plant Physiol. 157:574–586. doi: 10.1104/pp.111.178301 PMID: 21807887

8. Shi R, Sun YH, Li Q, Heber S, Sederoff R, Chiang VL. (2010) Towards a systems approach for lignin biosynthesis in *Populus trichocarpa*: transcript abundance and specificity of the monolignol biosynthetic genes. Plant Cell Physiol. 51:144–163. doi: 10.1093/pcp/pcp175 PMID: 19996151
9. Silber MV, Meimberg H, Ebel J (2008) Identification of a 4-coumarate: CoA ligase gene family in the moss, *Physcomitrella patens*. Phytochemistry. 69:2449–2456. doi: 10.1016/j.phytochem.2008.06.014 PMID: 18722632
10. Hu WJ, Kawaoka A, Tsai CJ, Lung J, Osakabe K, Ebinuma H, et al. (1998) Compartmentalized expression of two structurally and functionally distinct *4-coumarate:CoA ligase* genes in aspen (*Populus tremuloides*). Proc Natl Acad Sci. USA. 95:5407–5412. PMID: 9560289
11. Wagner A, Donaldson L, Kim H, Phillips L, Flint H, Steward D, et al. (2009) Suppression of 4-Coumarate-CoA Ligase in the Coniferous Gymnosperm *Pinus radiate*. Plant Physiol. 149:370–383. doi: 10.1104/pp.108.125765 PMID: 18971431
12. Kajita S, Katayama Y, Omori S (1996) Alterations in the biosynthesis of lignin in transgenic plants with chimeric genes for 4-coumarate:coenzyme A ligase. Plant Cell Physiol. 37:957–965. PMID: 8979396
13. Lee D, Meyer K, Chapple C, Douglas CJ (1997) Antisense suppression of 4-coumarate:coenzyme A ligase activity in *Arabidopsis* leads to altered lignin subunit composition. Plant Cell. 9:1985–1998. PMID: 9401123
14. Li L, Zhou Y, Cheng X, Sun J, Marita JM, Ralph J, et al. (2003) Combinatorial modification of multiple lignin traits in trees through multigene cotransformation. Proc Natl Acad Sci USA. 100:4939–4944. PMID: 12668766
15. Baur JA and Sinclair DA (2006) Therapeutic potential of resveratrol: the in vivo evidence. Nat Rev Drug Discov. 5:493–506. PMID: 16732220
16. Doré S (2005) Unique properties of polyphenol stilbenes in the brain: more than direct antioxidant actions; gene/protein regulatory activity. Neurosignals. 14:61–70. PMID: 15956815
17. Shankar S, Singh G, Srivastava RK (2007) Chemoprevention by resveratrol: molecular mechanisms and therapeutic potential. Front Biosci. 12:4839–4854. PMID: 17569614
18. Katsuyama Y, Funa N, Miyahisa I, Horinouchi S (2007) Synthesis of unnatural flavonoids and stilbenes by exploiting the plant biosynthetic pathway in *Escherichia coli*. Chem Biol. 14:613–621. PMID: 17584609
19. Wang Y, Yi H, Wang M, Yu O, Jez JM (2011) Structural and Kinetic Analysis of the Unnatural Fusion Protein 4-Coumaroyl-CoA Ligase::Stilbene Synthase. J Am Chem Soc. 133:20684–20687. doi: 10.1021/ja2085993 PMID: 22129213
20. He N, Zhang C, Qi X, Zhao S, Tao Y, Yang G, et al. (2013) Draft genome sequence of the mulberry tree *Morus notabilis*. Nat Commun. 4:2445–2445. doi: 10.1038/ncomms3445 PMID: 24048436
21. Liu X, Xiao G, Chen W, Xu Y, Wu J (2004) Quantification and Purification of Mulberry Anthocyanins with Macroporous Resins. J Biomed Biotechnol. 2004:326–331. PMID: 15577197
22. Jia ZS, Tang MC, Wu JM (1999) The determination of flavonoid contents in mulberry and their scavenging effects in superoxide radicals. Food Chem. 64:555–559.
23. Halliwell B (2007) Dietary polyphenols: Good, bad, or indifferent for your health? Cardiovasc Res. 73:341–347. PMID: 17141749
24. Xu W, Yu Y, Zhou Q, Ding J, Dai L, Xie X, et al. (2011) Expression pattern, genomic structure, and promoter analysis of the gene encoding stilbene synthase from Chinese wild *Vitis pseudoreticulata*. J Exp Bot. 62:2745–2761. doi: 10.1093/jxb/erq447 PMID: 21504880
25. Li J, Zhao AC, Wang XL, Zhang QY, Li QY, Jin XY, et al. (2011) Molecular cloning and tissues expression analysis of three actin genes from mulberry (*Morus alba* L.). Acta Agron Sin. 37:641–649.
26. Wang CH, Liu C, Liu J, Xiang W, Huang XZ, Xu L. (2014) Antioxidant Activity of Medicine Mulberry (*Morus nigra*) in Xinjiang. Scientia Silvae Sinicae. 8:53–59.
27. Yan Y, Li Z, Koffas MA (2008) High-Yield Anthocyanin Biosynthesis in Engineered *Escherichia coli*. Biotechnol Bioeng. 100:126–140. PMID: 18023053
28. Gaid MM, Scharnhop H, Ramadan H, Beuerle T, Beerhues L (2011) 4-Coumarate:CoA ligase family members from elicitor-treated *Sorbus aucuparia* cell cultures. J Plant Physiol. 168:944–951. doi: 10.1016/j.jplph.2010.11.021 PMID: 21330005
29. Hamberger B and Hahlbrock K (2004) The *4-coumarate:CoA ligase* gene family in *Arabidopsis thaliana* comprises one rare, sinapate-activating and three commonly occurring isoenzymes. Proc Natl Acad Sci USA. 101:2209–2214. PMID: 14769935
30. Lindermayr C, Möllers B, Fliegmann J, Uhlmann A, Lottspeich F, Meimberg H, et al. (2002) Divergent members of a soybean (*Glycine max* L.) 4-coumarate:coenzyme A ligase gene family. Eur J Biochem. 269:1304–1315. PMID: 11856365

31. Chang LW, Juang LJ, Wang BS, Wang MY, Tai HM, Hung WJ, et al. (2011) Antioxidant and antityrosinase activity of mulberry (*Morus alba* L.) twigs and root bark. Food Chem Toxicol. 49:785–790. doi: 10.1016/j.fct.2010.11.045 PMID: 21130832
32. Kumar A and Ellis BE (2003) 4-coumarate:CoA ligase gene family in *Rubus idaeus*: cDNA structures, evolution, and expression. Plant Mol Biol. 51:327–340. PMID: 12602864
33. Li Y, Kim JI, Pysh L, Chapple C (2015) Four isoforms of *Arabidopsis thaliana* 4-coumarate: CoA ligase (4CL) have overlapping yet distinct roles in phenylpropanoid metabolism. Plant Physiol. 169:2409–2421. doi: 10.1104/pp.15.00838 PMID: 26491147
34. Yang J, Chen F, Yu O, Beachy RN (2011) Controlled silencing of 4-coumarate: CoA ligase alters lignocellulose composition without affecting stem growth. Plant Physiol Biochem. 49:103–109. doi: 10.1016/j.plaphy.2010.10.004 PMID: 21094613
35. Jenkins ME, Suzuki TC, Mount DW (1997) Mount evidence that heat and ultraviolet radiation activate a common stress-response program in plants that is altered in the UV-B mutant of *Arabidopsis thaliana*. Plant Physiol. 115:1351–1358. PMID: 9414549
36. Guy L. de B and Michael RR (2001) Signals regulating multiple responses to wounding and herbivores. Crit Rev Plant Sci. 20:487–521.
37. Sutela S, Hahl T, Tiimonen H et al. (2014) Phenolic compounds and expression of *4CL* genes in silver birch clones and Pt4CL1a lines. PLoS One. doi: 10.1371/journal.pone.0114434
38. Soltani BM, Ehlting J, Hamberger B, Douglas CJ (2006) Multiple cis-regulatory elements regulate distinct and complex patterns of developmental and wound-induced expression of *Arabidopsis thaliana 4CL* gene family members. Planta. 224:1226–1238. PMID: 16738863
39. Jin H, Cominelli E, Bailey P, Parr A, Mehrtens F, Jones J, et al. (2000) Transcriptional repression by AtMYB4 controls production of UV-protecting sunscreens in *Arabidopsis*. EMBO J. 19:6150–6161. PMID: 11080161
40. Lin J, Lin C, Li Y, Wu M, Tsai M, Hsing YI, et al. (2013) Interaction of small RNA-8105 and the intron of IbMYB1 RNA regulates IbMYB1 family genes through secondary siRNAs and DNA methylation after wounding. Plant J. 75:781–794. doi: 10.1111/tpj.12238 PMID: 23663233
41. Checker VG, Khurana P (2013) Molecular and functional characterization of mulberry EST encoding remorin (MiREM) involved in abiotic stress. Plant Cell Reports 32: 1729–1741. doi: 10.1007/s00299-013-1483-5 PMID: 23942844
42. Bartels S, Anderson JC, González Besteiro MA, Carreri A, Hirt H, Buchala A, et al. (2009) MAP kinase phosphatase1 and protein tyrosine phosphatase1 are repressors of salicylic acid synthesis and SNC1-mediated responses in *Arabidopsis*. Plant Cell. 21:2884–2897. doi: 10.1105/tpc.109.067678 PMID: 19789277
43. Schroeder JI, Kwak JM, Allen GJ (2001) Guard cell abscisic acid signalling and engineering drought hardiness in plants. Nature 410:327–330. PMID: 11268200
44. Hauffe KD, Paszkowski U, Schulze-Lefert P, Hahlbrock K, Dangl JL, Douglas CJ. (1991) A parsley 4CL-1 promoter fragment specifies complex expression patterns in transgenic tobacco. Plant Cell. 3:435–443. PMID: 1840921
45. Beekwilder J, Wolswinkel R, Jonker H, Hall R, de Vos CH, Bovy A. (2006) Production of resveratrol in recombinant microorganisms. Appl Environ Microbiol. 72:5670–5672. PMID: 16885328
46. Watts KT, Lee PC, Schmidt-Dannert C (2006) Biosynthesis of plant-specific stilbene polyketides in metabolically engineered *Escherichia coli*. BMC Biotechnol. 6:22. PMID: 16551366
47. Zhang Y, Li SZ, Li J, Pan X, Cahoon RE, Jaworski JG, et al. (2006) Using unnatural protein fusions to engineer resveratrol biosynthesis in yeast and Mammalian cells. J Am Chem. 128:13030–13031.
48. Donnez D, Jeandet P, Clément C, Courot E (2009) Bioproduction of resveratrol and stilbene derivatives by plant cells and microorganisms. Trends Biotechnol. 27:706–713. doi: 10.1016/j.tibtech.2009.09.005 PMID: 19875185
49. Zhang Z, Jin J, Shi L (2008) Protective function of cis-mulberroside A and oxyresveratrol from *Ramulus mori* against ethanol-induced hepatic damage. Environ Toxicol Pharmacol. 26:325–330. doi: 10.1016/j.etap.2008.06.008 PMID: 21791383
